# The role of TLR9 on *Leishmania amazonensis* infection and its influence on intranasal LaAg vaccine efficacy

**DOI:** 10.1371/journal.pntd.0007146

**Published:** 2019-02-25

**Authors:** Juliana Elena Silveira Pratti, Alessandra Marcia da Fonseca Martins, Juliana Paiva da Silva, Tadeu Diniz Ramos, Joyce Carvalho Pereira, Luan Firmino-Cruz, Diogo Oliveira-Maciel, Thiago Soares de Souza Vieira, Leandra Linhares Lacerda, Andre Macedo Vale, Celio G. Freire-de-Lima, Daniel C. Oliveira Gomes, Elvira M. Saraiva, Bartira Rossi-Bergmann, Herbert Leonel de Matos Guedes

**Affiliations:** 1 Instituto de Biofísica Carlos Chagas Filho, Universidade Federal do Rio de Janeiro, Rio de Janeiro, RJ, Brazil; 2 Department of Immunology, Instituto de Microbiologia Paulo de Góes, Universidade Federal do Rio de Janeiro, Rio de Janeiro, RJ, Brazil; 3 Laboratório de Imunobiologia, Núcleo de Doenças Infecciosas/ Núcleo de Biotecnologia, Universidade Federal do Espírito Santo, ES, Brazil; 4 Núcleo Multidisciplinar de Pesquisa UFRJ–Xerém em Biologia (NUMPEX-BIO), Campus Duque de Caxias Professor Geraldo Cidade (Polo Avançado de Xerém), Universidade Federal do Rio de Janeiro, Duque de Caxias, RJ, Brazil; Universiteit Antwerpen, BELGIUM

## Abstract

*Leishmania (L*.*) amazonensis* is one of the etiological agents of cutaneous leishmaniasis (CL) in Brazil. Currently, there is no vaccine approved for human use against leishmaniasis, although several vaccine preparations are in experimental stages. One of them is Leishvacin, or LaAg, a first-generation vaccine composed of total *L*. *amazonensis* antigens that has consistently shown an increase of mouse resistance against CL when administered intranasally (i.n.). Since Toll-like receptor 9 (TLR9) is highly expressed in the nasal mucosa and LaAg is composed of TLR9-binding DNA CpG motifs, in this study we proposed to investigate the role of TLR9 in both *L*. *amazonensis* infection and in LaAg vaccine efficacy in C57BL/6 (WT) mice and TLR9^-/-^ mice. First, we evaluated, the infection of macrophages by *L*. *amazonensis in vitro*, showing no significant difference between macrophages from WT and TLR9^-/-^ mice in terms of both infection percentage and total number of intracellular amastigotes, as well as NO production. In addition, neutrophils from WT and TLR9^-/-^ mice had similar capacity to produce neutrophil extracellular traps (NETs) in response to *L*. *amazonensis*. *L*. *amazonensis* did not activate dendritic cells from WT and TLR9^-/-^ mice, analysed by MHCII and CD86 expression. However, *in vivo*, TLR9^-/-^ mice were slightly more susceptible to *L*. *amazonensis* infection than WT mice, presenting a larger lesion and an increased parasite load at the peak of infection and in the chronic phase. The increased TLR9^-/-^ mice susceptibility was accompanied by an increased IgG and IgG1 production; a decrease of IFN-γ in infected tissue, but not IL-4 and IL-10; and a decreased number of IFN-γ producing CD8^+^ T cells, but not CD4^+^ T cells in the lesion-draining lymph nodes. Also, TLR9^-/-^ mice could not control parasite growth following i.n. LaAg vaccination unlike the WT mice. This protection failure was associated with a reduction of the hypersensitivity response induced by immunization. The TLR9^-/-^ vaccinated mice failed to respond to antigen stimulation and to produce IFN-γ by lymph node cells. Together, these results suggest that TLR9 contributes to C57BL/6 mouse resistance against *L*. *amazonensis*, and that the TLR9-binding LaAg comprising CpG motifs may be important for intranasal vaccine efficacy against CL.

## Author summary

Leishmaniasis is a major neglected tropical disease, being responsible for more than 20 million deaths per year. The high mortality rate highlights the difficulties and ineffectiveness of the current prophylactic approaches and treatments currently available. Therefore, the development of an effective vaccine would be highly advantageous to circumvent these problems. Despite the many vaccines preparations that have been studied in the last few years, none have shown satisfactory efficacy to be approved for human use. Immune receptors, including the TLR family, are known to be important for host defense during parasitic infections, such as leishmaniasis, and also for vaccine efficacy. In this work, we investigate the role of TLR9 during Leishmania amazonensis infection in vaccinated and non-vaccinated mice. We used a C57BL/6 TLR9-/- mouse model and a first-generation vaccine preparation (LaAg) composed of a total lysate of *L. amazonensis*. We demonstrate that TLR9 is important for controlling leishmaniasis infection caused by L. amazonensis and is involved in the efficacy of the LaAg vaccine. These findings will certainly help in the development of a better vaccine against leishmaniasis.

## Introduction

Leishmaniasis is a group of chronic, non-contagious diseases caused by flagellate protozoa of the genus *Leishmania* [1]. *Leishmania (L*.*) amazonensis* is an etiological agent for a broad spectrum of leishmaniases in South American countries [2], including Brazil, where it is a causative agent of localized cutaneous leishmaniasis, diffuse cutaneous leishmaniasis and, rarely, visceral leishmaniasis [2]. Most cases of *L*. *amazonensis* infections in Brazil are concentrated in the north of the country (Amazon Forest Region). All medications used in the treatment of leishmaniasis are toxic and expensive. There are also difficulties in controlling the disease due to the great biological parasite diversity, the different clinical forms of the disease, including those severe forms resistant to chemotherapy, thus making prevention through vaccination the best strategy.

Currently, there is no vaccine approved for human use against leishmaniasis; however, several vaccine preparations are being studied. The Leishvacin® (or LaAg) vaccine is a first-generation vaccine composed of total proteins, lipids, carbohydrates, RNA and DNA of *L*. *amazonensis* and has been studied for years [3,4]. The efficacy of the mucosa as administration route of LaAg vaccine has already been tested in both oral and intranasal routes [5,6]. Studies show that intranasal administration of LaAg provides greater protection to BALB/c mice challenged with *L*. *amazonensis* and is more advantageous due to the easier application, and lower doses of antigen required as compared to the oral route. The protection achieved by intranasal immunization was accompanied by the development of a long-term immune memory and adaptive immunity [5,7].

Toll-like receptors (TLRs) are transmembrane proteins that recognize pathogen-associated molecular patterns (PAMPS) [8]. The TLRs play an important role during *Leishmania* infections. TLR9 recognizes unmethylated CpG DNA sequences, which are commonly found in bacteria and *Leishmania* [9], but not in mammalian cells where these sequences are normally methylated [10]. It has been shown that the activation of TLR9 promotes a host-protective response. For example, TLR9-dependent activation of dendritic cells (DCs) by DNA from *L*. *major* favors Th1 cell development and lesion resolution [11]. TLR9 signaling is essential for the innate natural killer (NK) cell response in murine cutaneous leishmaniasis caused by *L*.*major* [12]. Similarly, in visceral leishmaniasis caused by *L*. *major*, the activation of NK cell also requires TLR9 [13]. DC activation by *L*. *braziliensis* has also been shown to be dependent on TLR9 *in vitro* [14]. Furthermore, TLR9^-/-^ mice inoculated with *L*. *braziliensis* exhibited transiently increased lesion sizes and parasite burdens in comparison with those of control mice [14]. DNA from *L*. *mexicana* activates murine bone marrow-derived macrophages leading to the production of proinflammatory cytokines, such as TNF-α and IL-12, as well as the overexpression of mRNA for TLR9 [15].

Despite all this knowledge, our understanding of TLRs and cytoplasmic pattern recognition receptors (PRRs) that recognize and respond to *Leishmania* is rather limited [16]. In this study, we investigated the role of TLR9 during *L*. *amazonensis* infection in C57BL/6 (WT) mice and C57BL/6 TLR9^-/-^ mice, the adjuvant effect of the DNA containing CpG motifs present in LaAg vaccine and their efficacy when administered by the mucosal route, which presents high expression of TLR9 [17].

## Materials and methods

### Animals

C57BL/6 WT mice were acquired from Universidade Federal Fluminense (UFF) and Universidade Federal do Rio de Janeiro (Fundação BioRio). *Tlr9*^*−/−*^ mice in the C57BL/6 background were generated by and obtained from Dr. S. Akira (Osaka University, Japan). All animals were maintained in our own animal facility at UFRJ using sterilized bedding, filtered water and commercial feed *ad libitum*. Experimental groups consisted of C57BL/6 WT or TLR9^-/-^ mice at 6–8 weeks of age. The Health Sciences Center Ethics Committee of Federal University of Rio de Janeiro (Comissão de Ética no Uso de Animais do Centro de Ciências da Saúde da Universidade Federal do Rio de Janeiro) approved the animal use under the protocol number IBCCF 157.

### Parasites

The parasites used in this study were *L*. *amazonensis* (MHOM/BR/75/Josefa) originally isolated from human cutaneous leishmaniasis [18], maintained at 26°C in M199 medium (Sigma) containing 10% heat-inactivated fetal bovine serum (SFB, Cultilab) and hemin (5μg/ml, Sigma). To ensure infectivity, amastigotes were isolated from lesions of pre-infected BALB/c mice and promastigotes were only used until the fifth passage of the culture.

### Macrophage isolation

Macrophages were isolated from the peritoneal cavity of mice following the injection and withdrawal of RPMI (Gibco, NM, USA) medium. The cells were counted with Trypan blue and transferred to a 24 well plate at a concentration of 5×10^5^ cells/well, then incubated for 1 h at 37°C and 5% CO_2_ to allow the macrophages to adhere to the plate. Thereafter, the plate was washed with PBS three consecutive times to remove the non-adherent cells, and 400 μL RPMI with 10% FBS supplemented with glutamine, pyruvate, and non-essential amino acids was added. After 24 h, the wells were washed again with PBS to remove B1 lymphocytes and 300μL RPMI with 10% FBS was added.

### *In vitro* infection of macrophages

Macrophages (5×10^5^ cells/well) were infected with 2.5×10^6^ of stationary-phase *L*. *amazonensis* (ratio of 5:1). After 4h, the wells were washed with PBS three times and left at 37°C and 5% CO_2_. After 48 h, the supernatants were recovered and the plate was washed again with PBS three times. Finally, the plate was fixed and stained with a fast panoptic kit (Laborclin, RJ, Brazil). The infection was analyzed by optical microscopy (CX31, Olympus, Japan). One hundred (100) macrophages were counted per well and it was evaluated whether the macrophages were infected or not (percentage of infection) and the number of intracellular amastigotes in the infected macrophages were counted.

### NO quantification assay

After *in vitro* infection, the supernatants were collected and placed in a 96 well plate (100 μl/well) followed by Griess reagent (100 μl/well) (SIGMA). The plate was incubated for 10 min and the optical absorbance was read at 540 nm.

### Neutrophil isolation

Neutrophils were obtained from mouse bone marrow as previously described [19]. Briefly, bone marrow cell suspension obtained by femur and tibia flushing were centrifuged in a Percoll gradient (58%, 65% and 72% v.v.; GE Healthcare, Little Chalfont, UK), and 74.5 ± 2.59% Ly6G+ neutrophils were obtained through this method.

### NET quantification assay

Neutrophils (5×10^5^ cells/well) were either left untreated (Nil) or incubated with increasing amounts of promastigotes (1:1, 1:5) for 4 h at 35°C and 5% CO_2_. Culture supernatants were collected and extracellular DNA was analyzed using Quant-IT dsDNA Picogreen kit (Invitrogen). Fluorescence was detected using a SpectraMax Paradigm microplate reader (Molecular Devices). Data was presented as fold increase over control.

### Fluorescence microscopy

Neutrophils (1×10^5^) seeded on a poly-L-lysine-coated glass slide were incubated with CSFE (0.5 μM, Invitrogen) stained-promastigotes (1 parasite/1 neutrophil ratio) for 4 h at 35°C and 5% CO_2_. Cells were fixed with 4% formaldehyde, washed with PBS, blocked with mouse serum for 60 min, washed twice with PBS and blocked again with 4% PBS-BSA for 30 min at room temperature. The cells were treated with anti-histone H1 (1:400; EMD Millipore, Billerica, MA, USA), followed by anti-mouse AF 546-Mab 3864 (1:400; EMD Millipore) and DAPI (Sigma) for 60 min in each step. Confocal images were taken using a Leica DMI-8 microscope.

### *In vitro* infection of dendritic cells

Naïve mice were euthanised and spleens from WT and TLR9^-/-^ mice were removed and treated with ACK. 1×10^6^ cells from the spleens were cultivated with 5×10^6^ *L*. *amazonensis* promastigotes stained with CFSE, or spleen cells were incubated with medium alone, as a as control. After 24 h, cells were stained for CD11c (PerCP), MHCII (PE), and CD86 (APC) and analysed by flow cytometry. Cells were gated on CD11c+, and further analysed based on the expression of MHCII+ and CD86+, and finally infection of these cells was determined by CFSE- or CFSE+ expression.

### LaAg preparation

*Leishmania amazonensis* promastigote antigens (LaAg) were prepared as previously described [20]. Briefly, stationary-growth phase promastigotes were washed three times in PBS and subjected to three cycles of freezing and thawing. LaAg was lyophilized, stored at -20°C and reconstituted with PBS immediately prior to use. For quality control, protein content was quantified using the Smith method with the bicinchoninic acid protein kit (Sigma-Aldrich), expecting a protein level around 60% and SDS-PAGE was performed routinely as described before [3, 4]. The quantification of the DNA content was performed by ultraviolet spectrophotometry after DNA purification using the Kit Extractme Genomic DNA (Blirt DNA Gdansk). The protein content was 647 μg and the DNA content was 0.8 μg in 1 mg of LaAg.

### Immunization, infection challenge, hypersensitivity and evaluation of disease progression

Mouse immunization was carried out by instillation of 10 μg of LaAg in 20 μl PBS (10 μl per nostril) using a micropipette adapted with a polystyrene microtip. A booster dose was given 7 days later [3]. The control mice received just PBS. Seven days post-boost, the animals were infected in the right hind paw with 5×10^5^ *L*. *amazonensis* stationary phase promastigotes in 20 ul PBS. For hypersensitivity, the size of footpad was evaluated 18 h, 24 h, and 48 h after infection using a pachymeter and expressed as the difference between the thicknesses of infected and contralateral PBS-injected paws [20]. For evaluation of lesion growth, lesion sizes were measured once a week using a pachymeter and expressed as the difference between the thicknesses of infected and contralateral non-infected paws. The parasite load was determined at the end of the experiments, when the infected foot was skinned and individually homogenized in 1 ml PBS using a tissue grinder. Tissue debris were removed by gravity sedimentation for 5 min. The homogenates were then submitted to limiting dilution assay (LDA).

### Specific antibody production

Quantification of antibody production was carried out using ELISA (Goat Anti-Mouse IgM-UNLB:Cat. No.1021-01, Goat Anti-Mouse IgG Fc-UNLB:Cat. No. 1033–01, Goat Anti-Mouse IgG1-UNLB:Cat. No. 1071–01, SouthernBiotec). First, total *Leishmania amazonensis* antigen (LaAg) diluted in PBS (5 μg/mL) was added to the plate for coating overnight. On the second day, the content was discarded and the plate was blocked with Block Buffer (PBS with 5% heat-inactivated fetal bovine serum (HIFCS, GIBCO Laboratories, Grand Island, NY, USA) and 0.05% Tween 20) for 1 h. The plate was then washed three times with Wash Buffer (PBS with 0.05% Tween 20) and the samples were diluted in Block Buffer and added to the plate. After 1 h, the plate was washed five times with Wash Buffer and the secondary antibody specific for each isotype of interest (one for each plate) was added. After 1 h, the plate was washed seven times with Wash Buffer and TMB was added. Finally, the reaction was blocked with HCl.

### Cytokine quantification (in situ and from supernantants of lymphocyte proliferation)

For *in situ* production [21], infected paws were isolated as mentioned above. The paw homogenates were centrifuged (10 min, 20,000 × g, 4°C) and the supernatants were collected. For antigen stimulation, lesion-draining popliteal lymph nodes were excised 72 h post infection and single-cell suspensions were prepared. The cells were plated at a concentration of 1×10^6^ cells/ml and were stimulated with 50 μg/mL of *Leishmania major* antigens (LmAg) for 72 h at 37°C with 4% CO_2_ (For *in vitro* assay, we used LmAg instead of LaAg since the latter induces apoptosis [4]). IFN-γ, IL-10 and IL-4 cytokines were quantified in the supernatant by ELISA following the manufacturer’s instructions (R&D Systems).

### Flow cytometry

Lymph node cells were cultured for 4 h at 37°C in the presence of PMA (20 ng/ml), Ionomycin (1 μg/ml) and brefeldin A (Sigma-Aldrich). The cells were surface stained with Anti-CD3-PerCP, anti-CD8-APC-Cy7 and anti-CD4-PE-Cy7 (Biolegend) and were fixed and permeabilized for 1 h using the Foxp3/Transcription Factor Fixation/Permeabilization Kit (e-Bioscience, Santa Clara, USA). Intracellular cytokine staining was performed with anti-IFN-γ-APC and IL10-Pe (Biolegend). At least 10,000 gated CD4^+^ lymphocyte events were acquired in a BD FACSCanto™ II (BD Biosciences New Jersey, USA) and the data was analysed with FlowJo X software.

### Statistical analysis

Experiments were performed two or three independent times, and the result of one representative experiment is shown. The lesion sizes caused by the infection were statistically analyzed by Two-way ANOVA following Bonferroni’s *post hoc* test. The results provided in the remaining figures were tested by Student’s *t*-test. We used GraphPad Prism v.5 software, and the results were considered significant when *P* ≤ 0.05.

## Results

### Evaluation of TLR9 role during *in vitro* infection of macrophages by *L*. *amazonensis*

**Fig 1:**
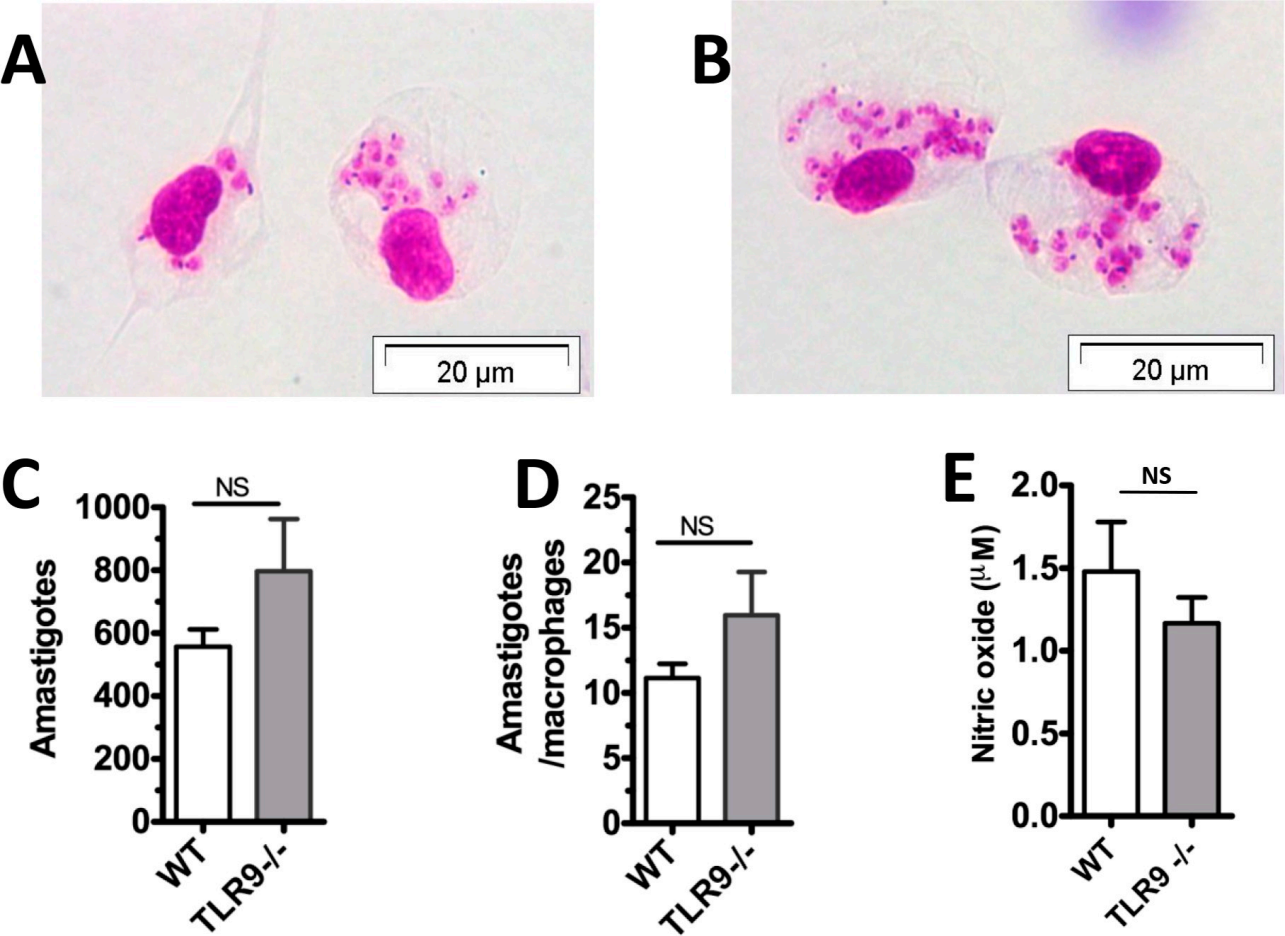
Evaluation of *in vitro* infection of TLR9^-/-^ macrophages by *L*. *amazonensis*. Macrophages (5×10^5^ cells/well) obtained from peritoneum of WT C57BL/6 and TLR9^-/-^ mice were plated and infected with 2.5×10^6^ *L*. *amazonensis* (1:5). After 4 h, the plates were washed and the cells were analyzed. The plate was stained 48 h after the infection with fast panoptic kit. *Leishmania* were counted using an optical microscope, Olympus CX31, at 100x magnification; photos weretaken using an inverse fluorescence Olympus BX51 microscope at 40x magnification with the program Cell F 3.1. **A.** C57BL/6 WT macrophages infected with *L*. *amazonensis*. **B.** TLR9^-/-^ macrophages infected with *L*. *amanonensis*. **C.** Total amastigotes. **D.** Ratio of intracellular amastigotes per 100 macrophages. **E.** NO quantification. Triplicate average of one experiment (*P* ≤ 0.05). Results representative of three independent experiments.

To assess the *L*. *amazonensis* infection profile of macrophages from C57BL/6 (WT) and TLR9^-/-^ mice *in vitro*, peritoneal cells were infected and analyzed after 48 h as described in the methodology. Using light microscopy, the infection of WT macrophages (Fig 1A) and TLR9^-/-^ macrophages (Fig 1B) was evaluated, and a similar infection profile was observed between both groups. The analysis of the total number of amastigotes (Fig 1C) and the ratio of amastigote/macrophage (Fig 1D) also presented similar results. Likewise, the production of nitric oxide (NO) showed no significant difference between WT and TLR9^-/-^ macrophages (Fig 1E).

### TLR9 is not involved in NET production stimulated by *L*. *amazonensis*

**Fig 2:**
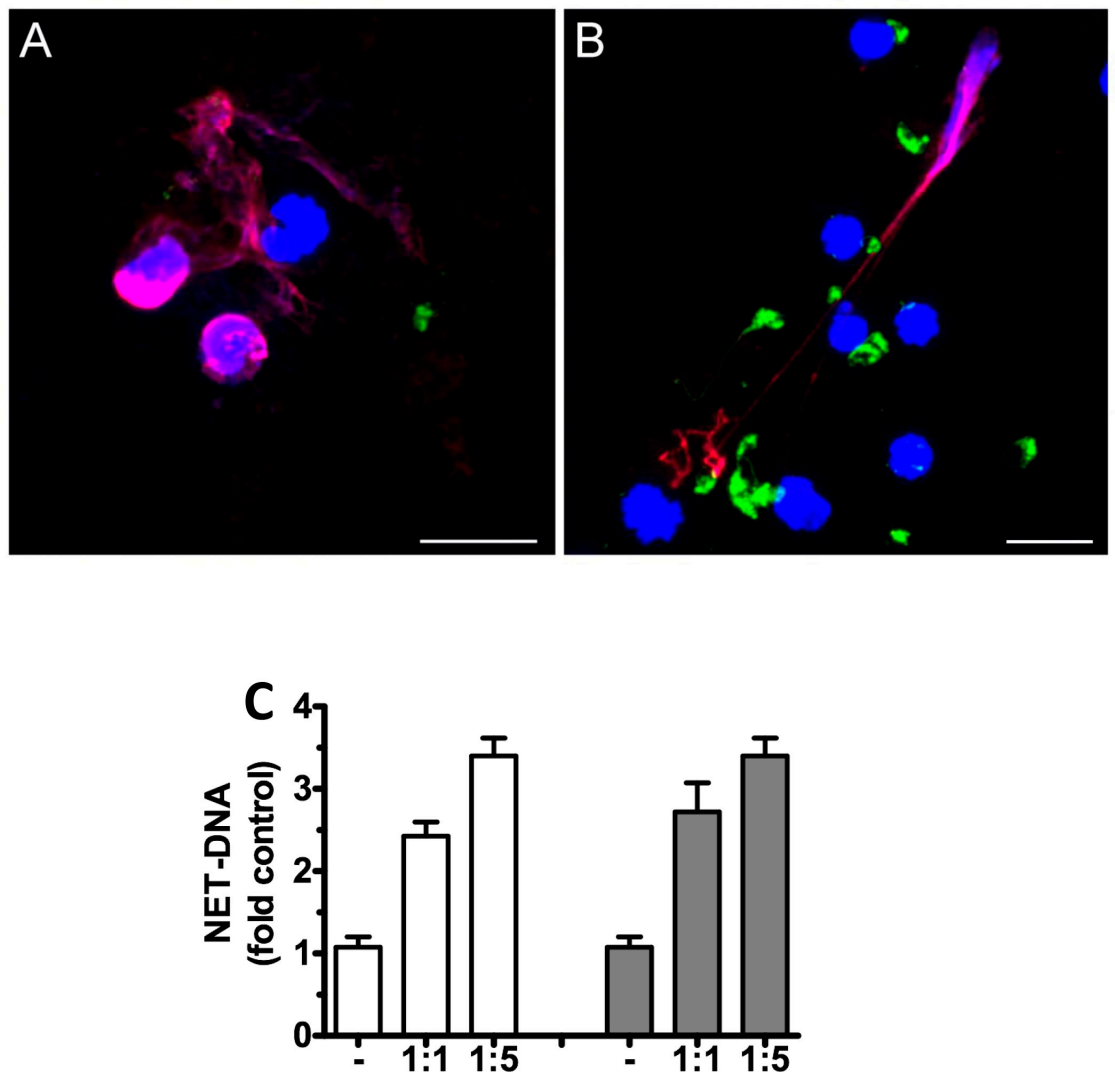
Neutrophil extracellular traps (NETs) induction by *L*. *amazonensis* in WT and TLR9 ^-/-^ neutrophils. Neutrophils (1×10^5^) were obtained from bone marrow and incubated with CSFE stained-promastigotes (La, green) at 1:5 neutrophils/parasite ratio for 4 h. Cells were then fixed and stained for anti-DNA/Histone H1, followed by the anti-mouse antibody (red) and DAPI (blue). **A.** C57BL/6 WT neutrophils interacting with *L*. *amazonensis*. **B.** TLR9^-/-^ neutrophils interacting with *L*. *amazonensis* (Bar = 10 μm). **C.** Bone-marrow derived neutrophils (5×10^5^ cells/well) from WT (white bars) or TLR9^-/-^ (gray bars) mice were either left untreated or incubated with increasing doses of *L*. *amazonensis* promastigotes (1:1, 1:5) and NET formation was measured as the release of dsDNA measured as fold increase over control (Nil). n = 4 out of two independent experiments.

The formation of neutrophil extracellular traps (NETs) derived from WT and TLR9^-/-^ bone marrow neutrophils in response to *L*. *amazonensis* (Fig 2) was assessed. NET release was analysed using the immunofluorescence microscopy after staining for anti-DNA/Histone H1. No difference was observed in the NET release by WT (Fig 2A) and TLR9^-/-^ neutrophils (Fig 2B) stimulated with *L*. *amazonensis*. The release of dsDNA using the DNA picogreen assay of neutrophils from WT and TLR9^-/-^ neutrophils was also measured (Fig 2C), and, again, no difference was observed. These results suggest that TLR9 does not participate in NET induction by *L*. *amazonensis* infection *in vitro*.

### *Leishmania amazonensis* infection does not activate dendritic cells *in vitro*

**Fig 3:**
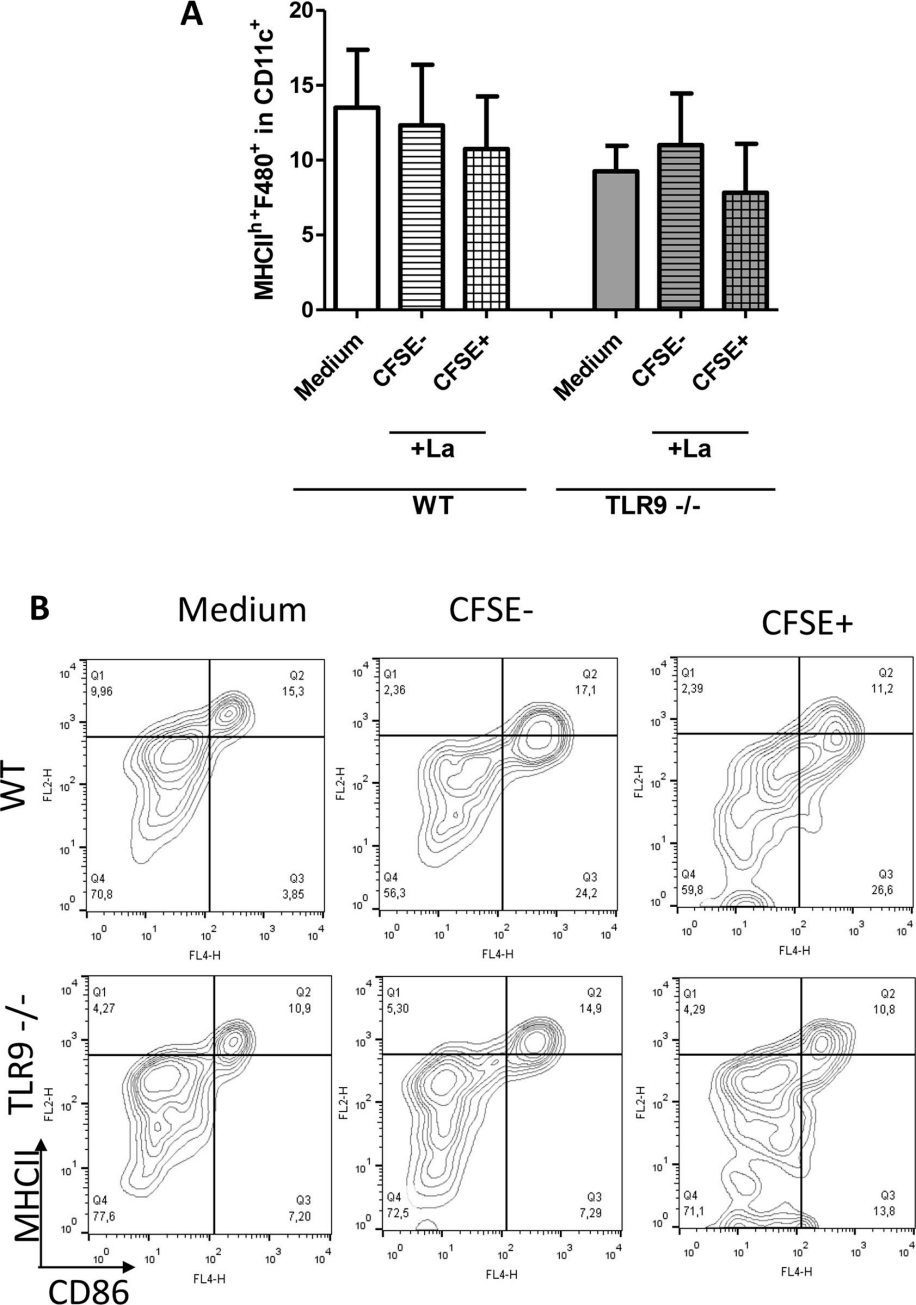
*In vitro* infection of dendritic cells by *Leishmania amazonensis*. Spleen cells (1×10^6^) from C57BL/6 WT and TLR9^-/-^ mice were incubated with 5×10^6^ *L*. *amazonensis* promastigotes stained with CFSE, or incubated with medium as a control. After 24 h, cells were stained for CD11c+ (PerCP), MHCII (PE), and CD86 (APC). Cells were gated on CD11c+ expression, followed by the expression of MHCII and CD86. A: Percentage of double positive MHCII^hi^ and CD86^+^ dendritic cells from WT and TLR9^-/-^ mice (mean ± SD; n  =  4). B: Representative of dot plots.

Based on the role of TLR9 in the activation of dendritic cells, the percentage of the DC population, through the expression of MHCII and CD86, from the spleen of WT and TLR9^-/-^ mice was evaluated by flow cytometry following *L*. *amazonensis* infection. As was demonstrated before [22, 23, 24], *Leishmania amazonensis* does not have the ability to activate DC, as can be observed in the similar percentages of double positive MHCII^hi+^CD86^+^ in WT, regardless of whether the cells were infected or not, as identified through CFSE expression from parasites, or a non-infected medium control was used (Fig 3A). In TLR9^-/-^, a similar profile was observed (Fig 3A). There was no difference in CFSE+ DCs between WT and TLR9^-/-^ samples, indicating that there is no difference in phagocytosis of *L*. *amazonensis* (SF3).

### *In vivo* infection of TLR9^-/-^ mice by *L*. *amazonensis*

**Fig 4:**
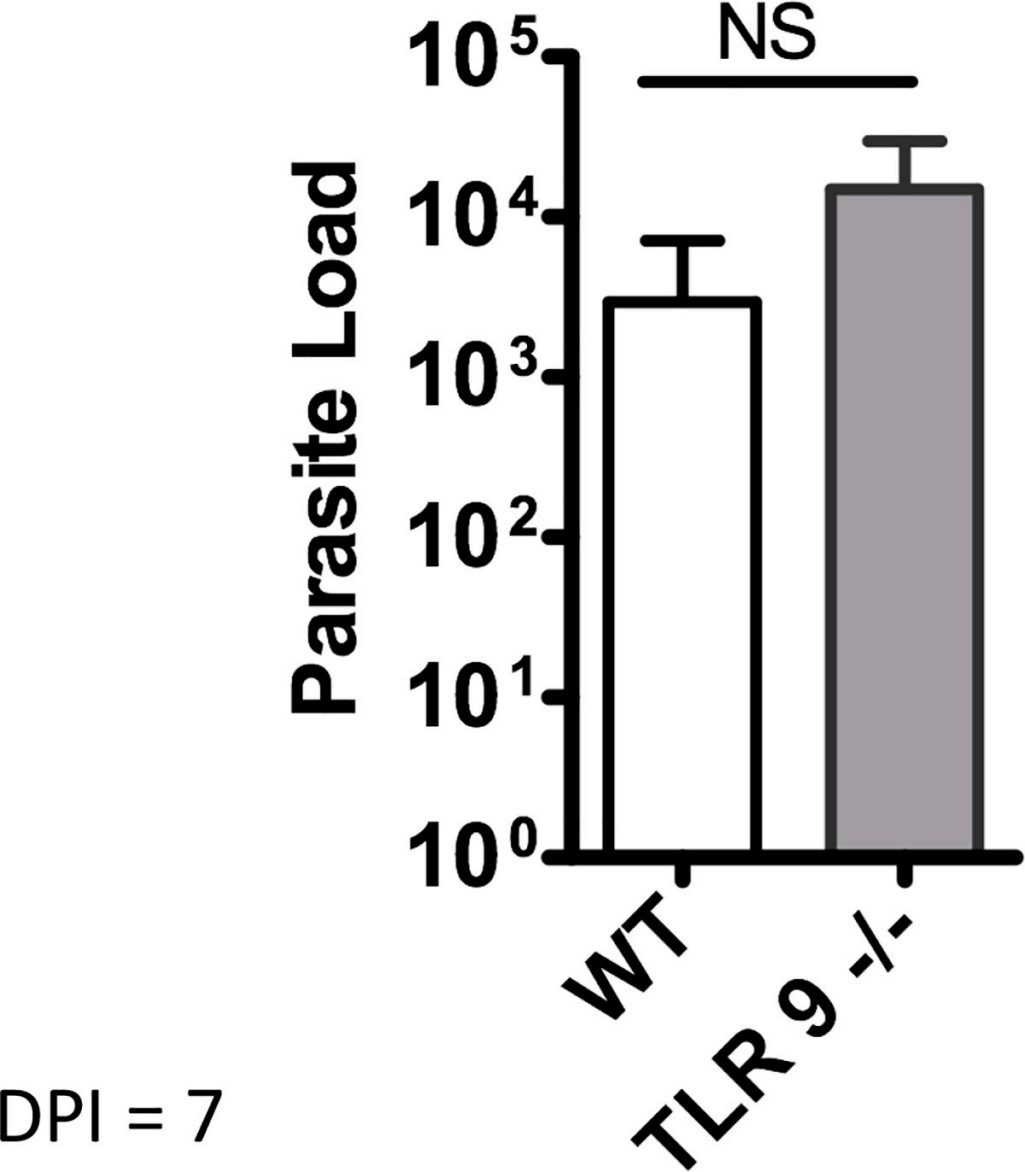
Evaluation of *in vivo L*. *amazonensis* infection in TLR9^-/-^ mice. C57BL/6 WT and TLR9^-/-^ mice were infected in the right hind paw with 5×10^5^ *L*. *amazonensis* promastigotes (Josefa strain). The parasite load was measured after 7 days post-infection and expressed as the mean number of parasites per paw (mean ± SD; n  =  4–5) *P ≤ 0.05. Results representative of two independent experiments.

**Fig 5:**
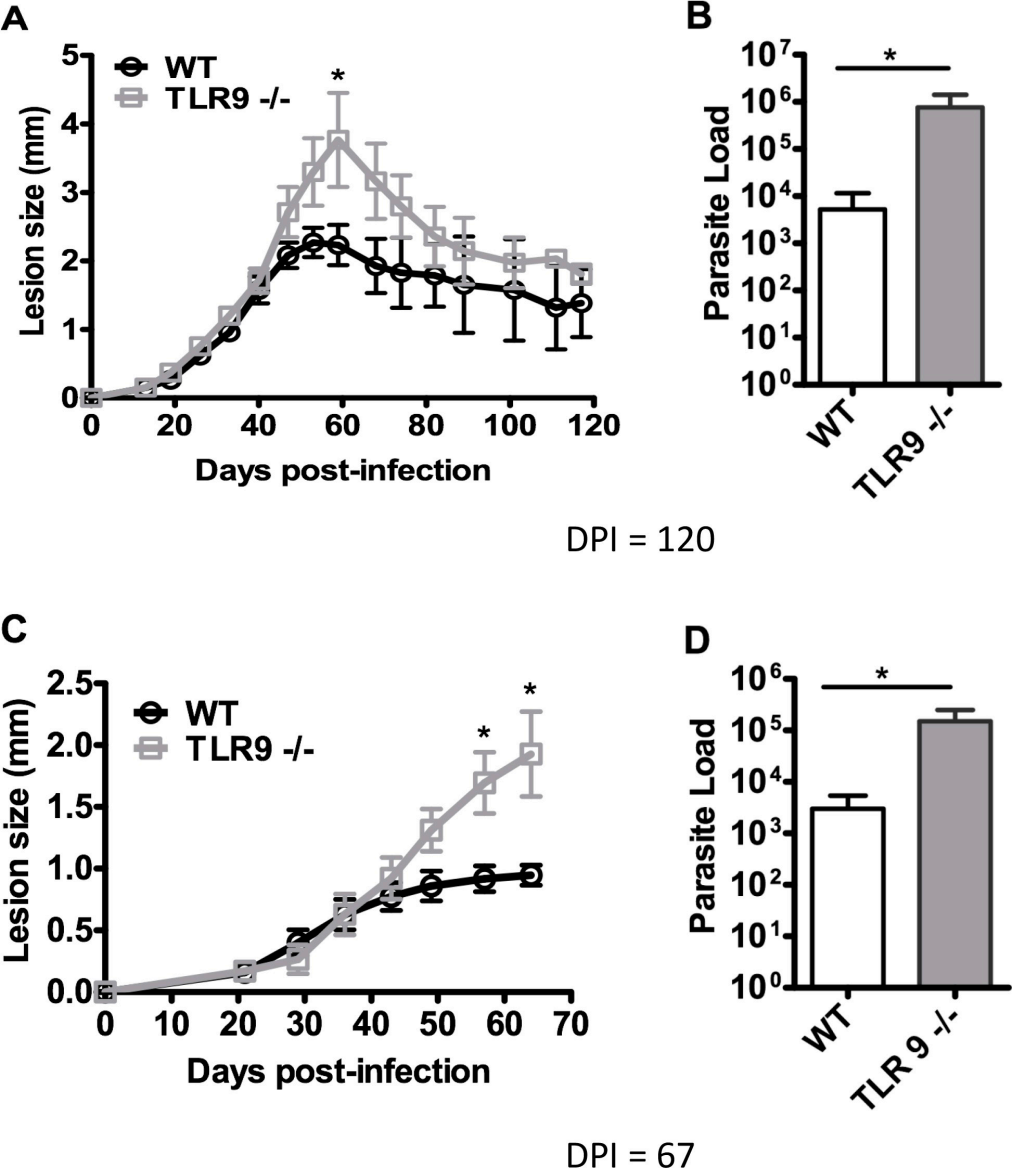
Evaluation of *in vivo* infection by *L*. *amazonensis*. C57BL/6 WT and TLR9^-/-^ mice were infected in the right hind paw with 5×10^5^ *L*. *amazonensis* promastigotes (Josefa strain). **A, C.** The lesion sizes were measured on the indicated days and expressed as the difference of thickness between non-infected and infected paws. **B.** Parasite load was measured after 120 days of infection (dpi) or **D**. 67 dpi and expressed as the mean number of parasites per paw (mean ± SD; n  =  4–5) *P ≤ 0.05. Results representative of three independent experiments.

To evaluate the role of TLR9 during *in vivo* infection, WT and TLR9^-/-^ mice were infected with *L*. *amazonensis*. No difference was observed in the parasite load 7 days post-infection (dpi) (Fig 4) indicating the absence of an early response dependent on TLR9. Lesion size of infected mice was monitored weekly which showed a similar progression in both groups until 42 dpi (Fig 5A). After which time, the lesion size in TLR9^-/-^ mice increased significantly more, with a peak around 60 dpi, followed by a partial resolution of the lesion with chronic parasite persistence in both groups until the last day (Fig 5A and 5B). Interestingly, although presenting similar lesion sizes at 120 dpi, the parasite load was significantly higher in TLR9^-/-^ mice (Fig 5B). In another set of experiments, mice lesions were monitored until the peak of infection (67 dpi), confirming the higher lesion size in infected TLR9^-/-^ mice (Fig 5C). The parasite load evaluated at 67 dpi in these groups was also significantly higher in TLR9^-/-^ mice when compared with WT mice (Fig 5D).

### Increase of specific antibody production on TLR9^-/-^ mice

**Fig 6:**
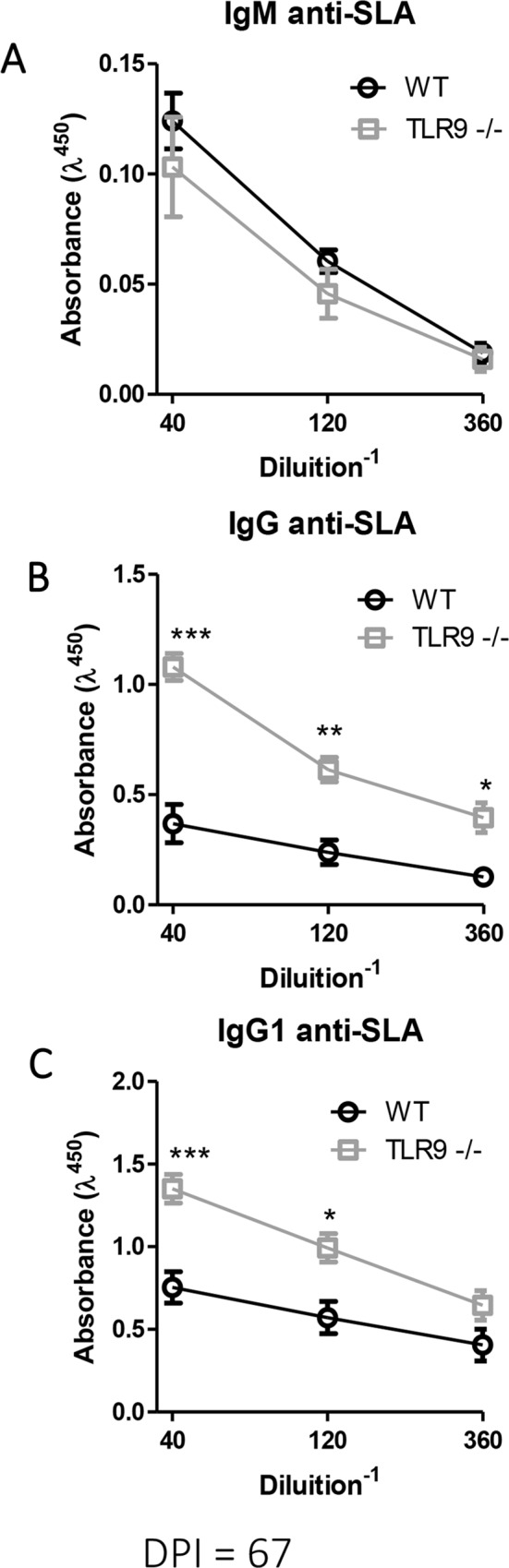
Evaluation of serum immunoglobulins in WT and TLR9^-/-^ mice. After 67 days of infection, sera of infected C57BL/6 WT and TLR9^-/-^ mice were evaluated to determine the levels of **A.** IgM, **B.** IgG and **C.** IgG1. (mean ± SD; n  =  4–5) ***P<0.001; **P<0.01; *P<0.05, obtained from Two-way Anova test with Bonferroni’s post-test. Results representative of two independent experiments.

Production of immunoglobulins by both WT and TLR9^-/-^ mice at 67 dpi was assessed by ELISA. TLR9^-/-^ mice produced higher amounts of IgG and IgG1 (Fig 6B and 6C) compared with WT mice, but there was no difference in IgM levels (Fig 6A). These results suggest an involvement of IgGs in the increase of infection susceptibility to *L*. *amazonensis* by TLR9^-/-^ mice.

### Decrease of interferon-gamma *in situ* production in TLR9^-/-^ mice

**Fig 7:**
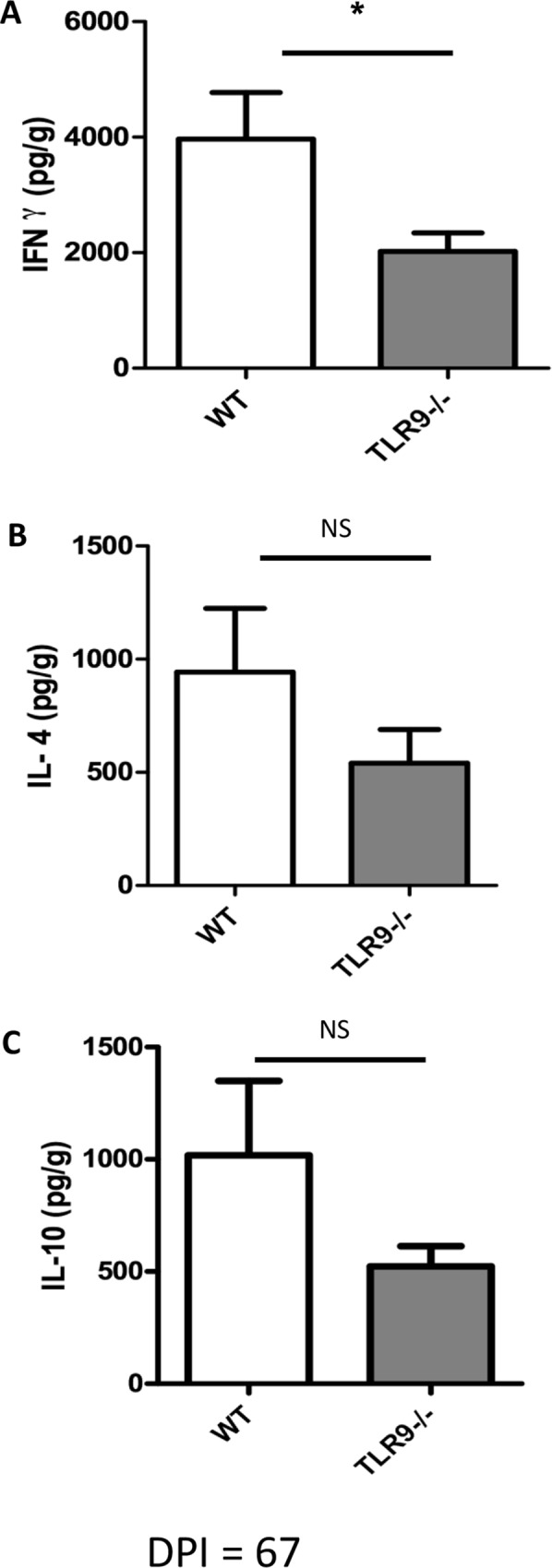
Evaluation of cytokine production in infected mice paws. After 67 days of infection, the infected C57BL/6 WT and TLR9^-/-^ mice were euthanized, the paw homogenates were centrifuged and the supernatants were collected for cytokine quantification by ELISA. **A.** IFN-γ **B.** IL-4 **C.** IL-10 **D.** TGF-β. **(**Mean ± SD; n = 4–5); (*) P ≤ 0.05. Results representative of two independent experiments.

In order to further identify possible causes of the differences in lesion size and parasite load at the peak of infection between the groups, the production of cytokines in mice paws at 67 dpi was also assessed by ELISA. The results showed a decrease in IFN-γ production by TLR9^-/-^ mice in comparison with WT mice (Fig 7A). However, the production of other cytokines, such as IL-4 and IL-10, showed no significant difference between the two groups (Fig 7B and 7C).

### Decreased number of IFN-γ producing CD8^+^ T cells in TLR9^-/-^ mice

**Fig 8:**
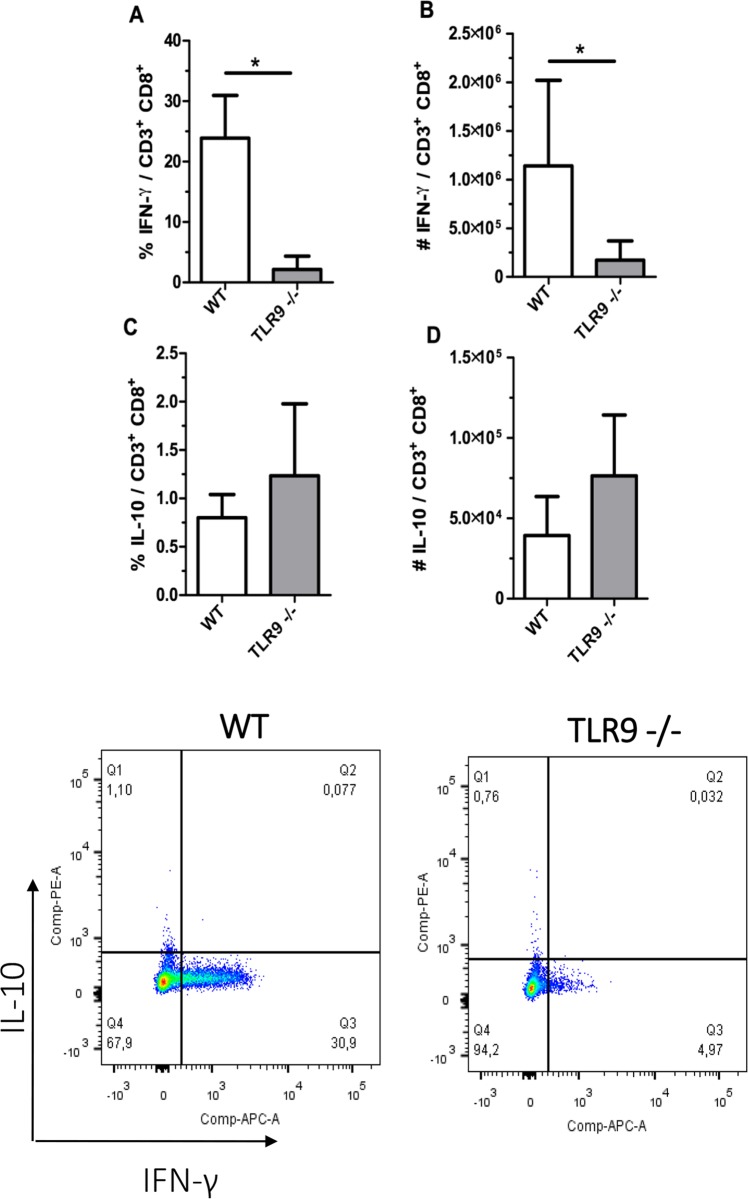
IFN-γ produced by CD8^+^ T cells present in the draining lymph node. Lymph node cells isolated from mice after 67 days of infection were cultured for 4 h in a solution with 20 ng/ml of PMA, 1μg/ml Ionomycin and brefeldin A. Cells were surface stained with anti-CD3-PerCP and anti-CD8-APC-CY7 and evaluated by flow cytometry (FACSCanto II BD). CD8^+^ T producing IFNγ^+^ A. percentage B. number of cells. CD8^+^ T producing IL-10^+^ C. percentage D. number of cells. Dot plot of CD8^+^ T cell gating strategy. (Mean ± SD; n = 4–5); (*) P ≤ 0.05. Results representative of two independent experiments.

**Fig 9:**
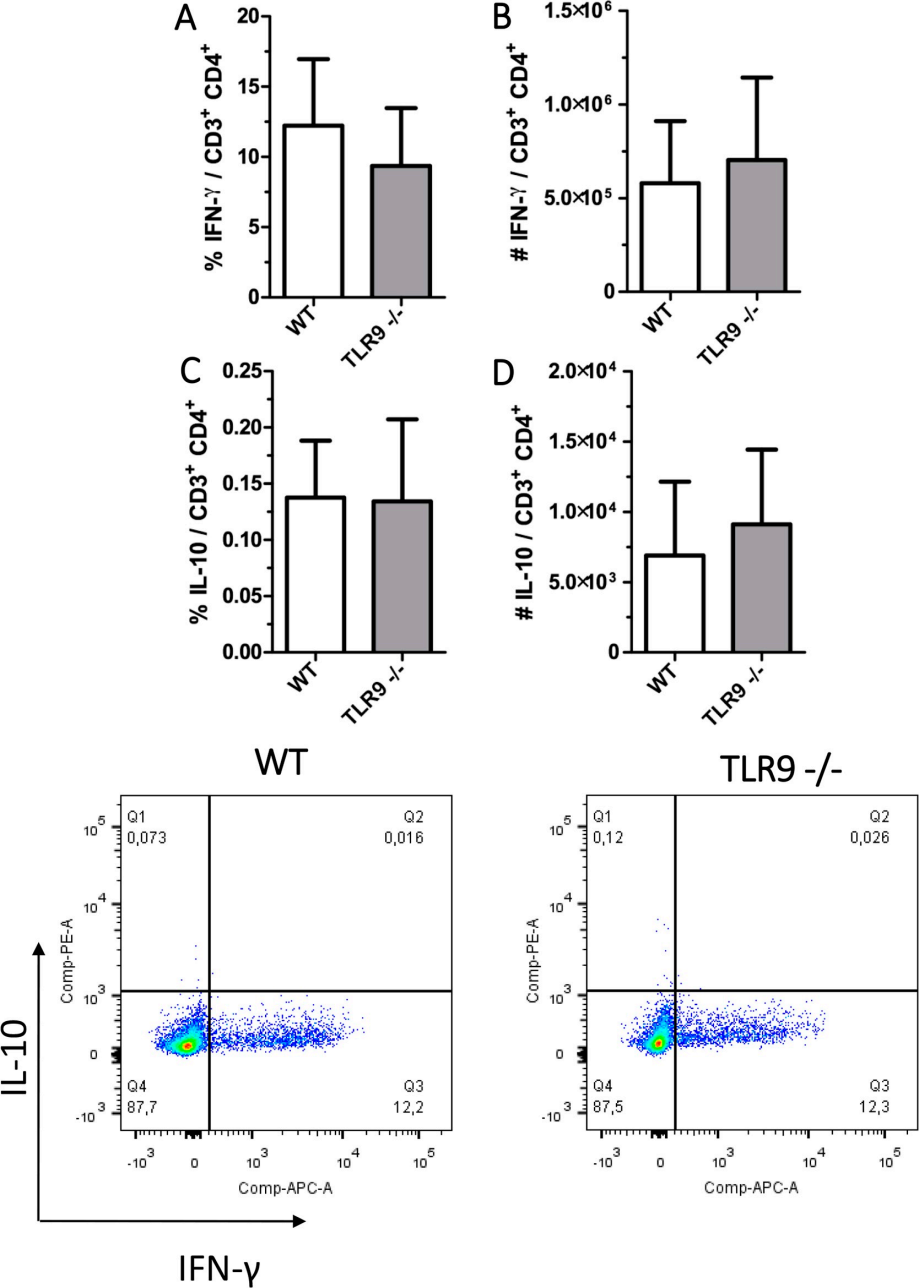
IFN-γ produced by CD4^+^ T cells present in the draining lymph node. Lymph node cells isolated from mice after 67 days of infection were cultured for 4 h in a solution with 20 ng/ml of PMA, 1μg/ml Ionomycin and brefeldin A. Cells were surface stained with anti-CD3-PerCP and anti-CD4-PE-Cy7 and evaluated by flow cytometry (FACSCanto II BD). **A.** CD4^+^ T IFN-γ^+^ **B.** CD4^+^ T IL-10^+^ **C.** Dot plot of CD4^+^ T staining gated on. Mean ± SD (n = 4–5); (*) P ≤ 0.05. Results representative of two independent experiments.

Due to the difference observed in IFN-y in the paw, flow cytometry was used to evaluate the percentage of IFN-γ produced by CD4^+^ T cells and CD8^+^ T cells present in the draining lymph node at 67 dpi. The results demonstrated a decrease in the percentage and number of IFN-γ producing CD8^+^ T cells in TLR9^-/-^ mice when compared with WT mice (Fig 8A and 8B). The percentage and numbers of IL-10-producing CD8^+^ T cells was not significantly different between the two groups (Fig 8C and 8D). The same cytokines were also evaluated in CD4^+^ T cells and the results were similar between both groups (Fig 9A–9D).

### LaAg vaccine protection was partially dependent on TLR9 activation

**Fig 10:**
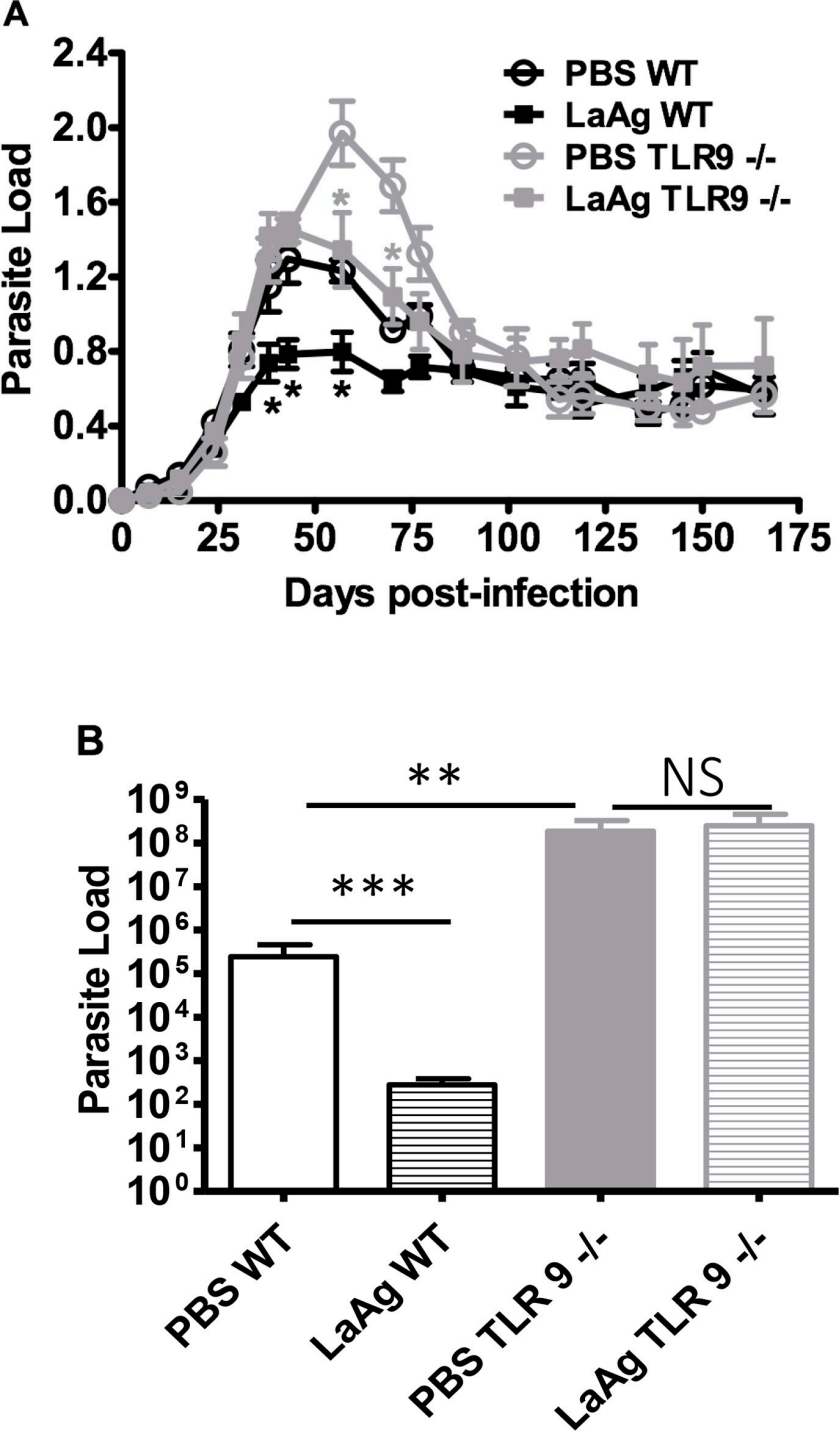
Efficacy of the LaAg vaccine given by intranasal route in the profile of *L*. *amazonensis* infection in WT and TLR9^-/-^ mice. C57BL/6 WT and TLR9^-/-^ mice were immunized by nasal instillation with 10 μg LaAg, using PBS as vehicle. Animals in the control group received only PBS. Immunization was performed in two doses, with a 7 day interval between each dose. One week after the 2nd dose, each animal was infected in the right hind paw with 5×10^5^ *L*. *amazonensis* promastigotes (Josefa strain). **A.** Lesion sizes were measured on the indicated days and expressed as the difference of thickness between non-infected and infected paws. **B.** Parasite load was measured after 166 days of infection and expressed as the mean number of parasites per paw (mean ± SD; n  =  4–5) *P ≤ 0.05 ***P<0.001; **P<0.01. Results representative of two independent experiments.

**Fig 11:**
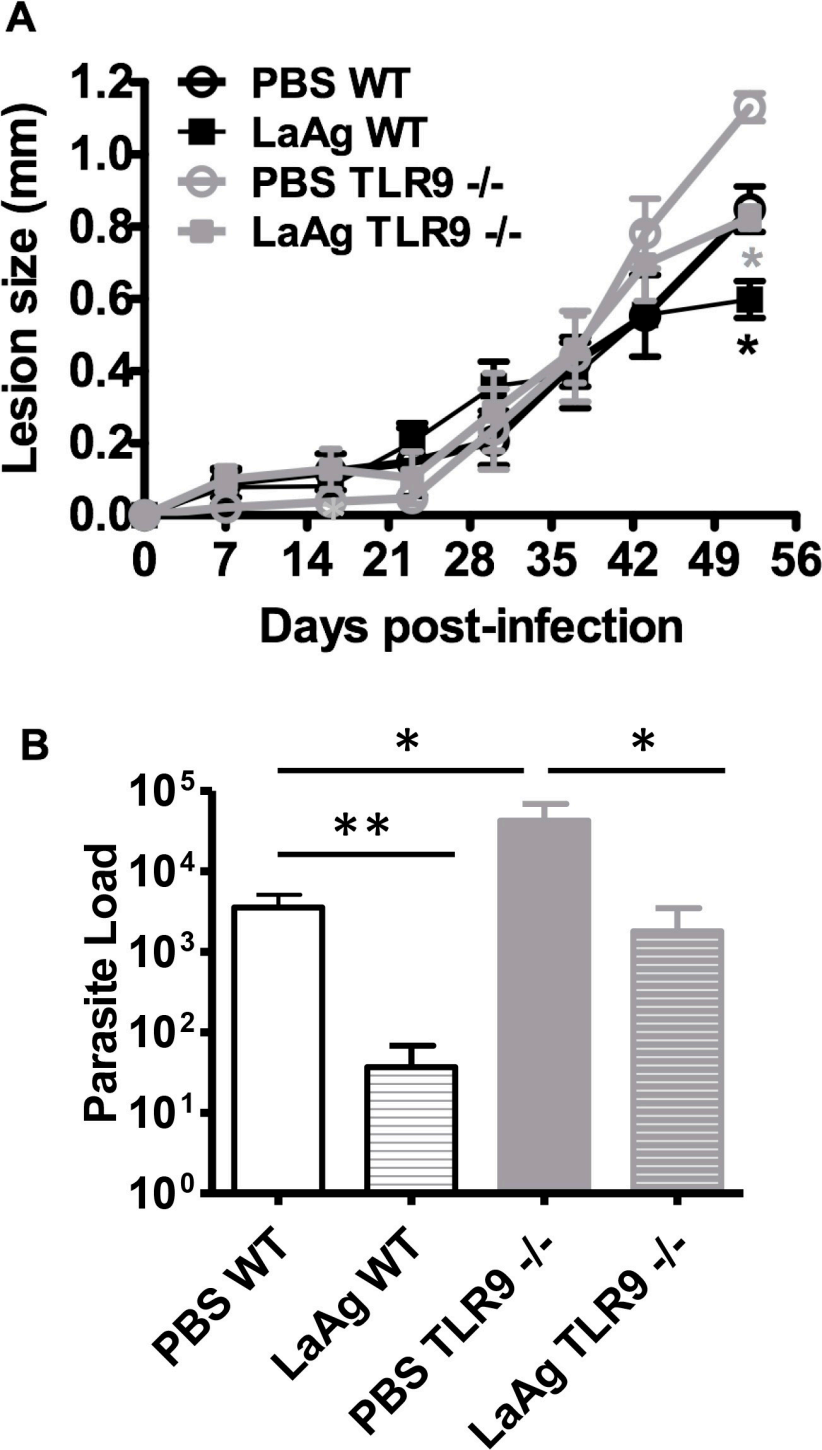
Efficacy of the LaAg vaccine given by the intranasal route at the peak of *L*. *amazonensis* infection in WT and TLR9^-/-^ mice. C57BL/6 WT and TLR9^-/-^ mice were immunized by nasal instillation with 10 μg LaAg using PBS as vehicle. Animals in the control group received only PBS. Immunization was performed in two doses, with a 7 day interval between each dose. One week after the 2nd dose, each animal was infected in the right hind paw with 5×10^5^ *L*. *amazonensis* promastigotes (Josefa strain). **A.** Growth curve of lesion size over course of infection. **B.** Parasite load of the infected paws determined at the end of the experiment by the LDA assay. (Mean ± SD;n = 4–5); (*) P ≤ 0.05 **P<0.01. Results representative of two independent experiments.

It is known that TLR9 is highly expressed in nasal mucosa [25] and that the LaAg vaccine has DNA in its composition [4]. Our results suggest that TLR9 participates in the induction of adaptive immune response against *L*. *amazonensis*, which lead us to consider the hypothesis that the recognition of the LaAg DNA by TLR9 in the mucosa could act as an adjuvant, improving LaAg vaccine. Thus, to assess whether there would be a relationship between the effectiveness of the LaAg vaccine and the activation of TLR9, the profile of *L*. *amazonensis* infection in LaAg-vaccinated WT and TLR9^-/-^ mice was investigated (Figs 10A and 11A).

In the peak of infection, vaccinated TLR9^-/-^ mice partially controlled the lesion in comparison with control TLR9^-/-^ mice injected with PBS (Figs 10A and 11A). Vaccinated TLR9^-/-^ mice showed no difference in relation to PBS WT mice, however, they demonstrated larger lesions in comparison with the LaAg-vaccinated WT mice (Figs 10A and 11A). The parasite loads at 52 dpi suggest that the LaAg vaccine efficacy is partially dependent on TLR9 activation in the peak of infection (Fig 11B). This dependency was more evident at 166 dpi, as vaccinated TLR9^-/-^ mice were unable to control parasite burden (Fig 10A and 10B).

### Protection failure of vaccinated TLR9^-/-^ mice was associated with reduction of hypersensitivity response and IFN-γ production by Ag-stimulation

**Fig 12:**
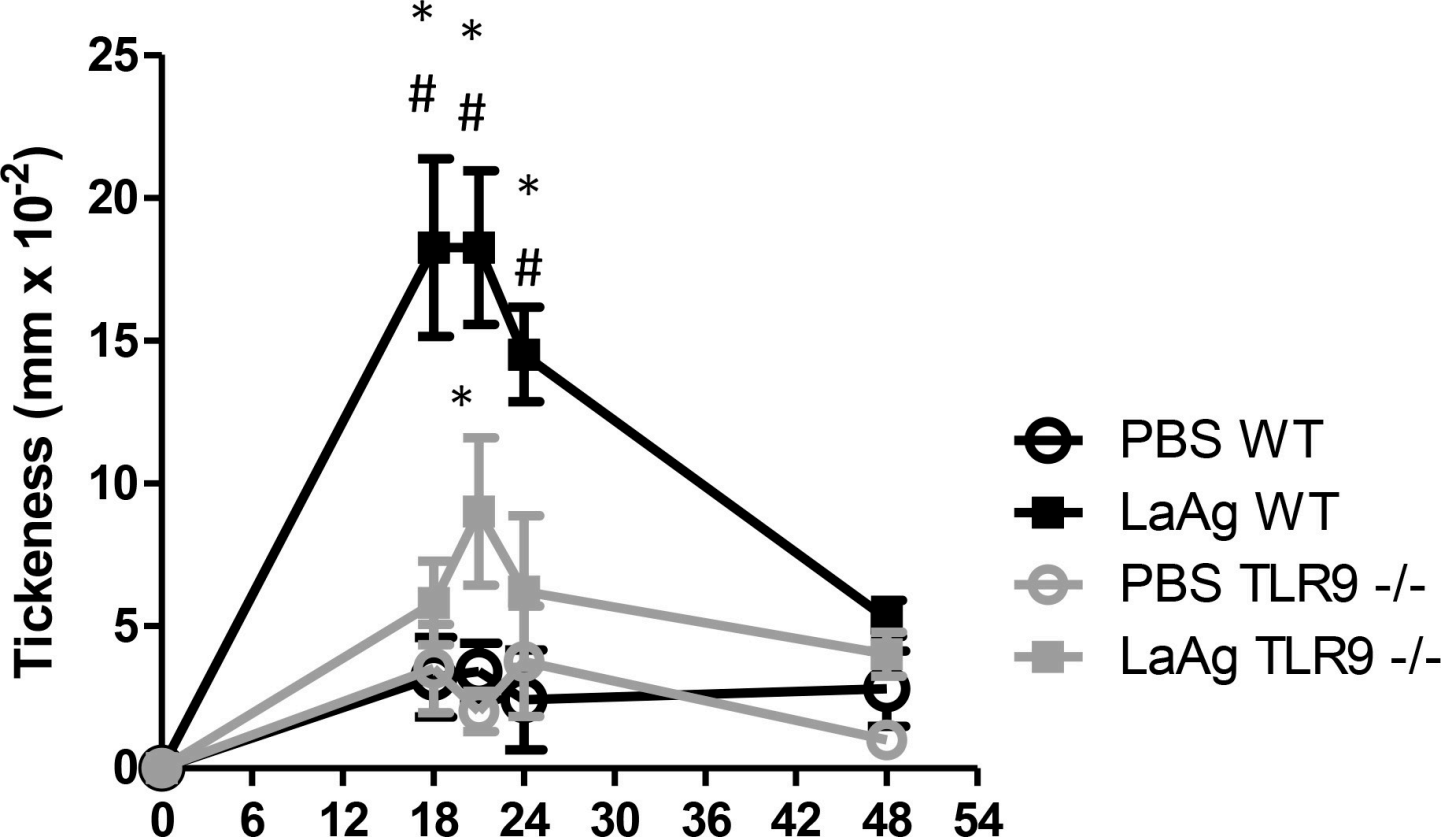
TLR9^-/-^ mice failed to induce delayed hypersensitivity response. Mice were vaccinated with LaAg on days -14 and -7 before infection, as described. Controls received PBS alone and the animals were then challenged with 5×10^5^ *L*. *amazonensis* in the paw. The kinetics of the hypersensitivity response was scored thereafter as the difference between infected and non-infected paw thicknesses. (Means ± SD; n = 5) *P≤0.01 in relation to PBS controls. # in relation to LaAg vaccinated TLR9^-/-^. Results representative of two independent experiments.

**Fig 13:**
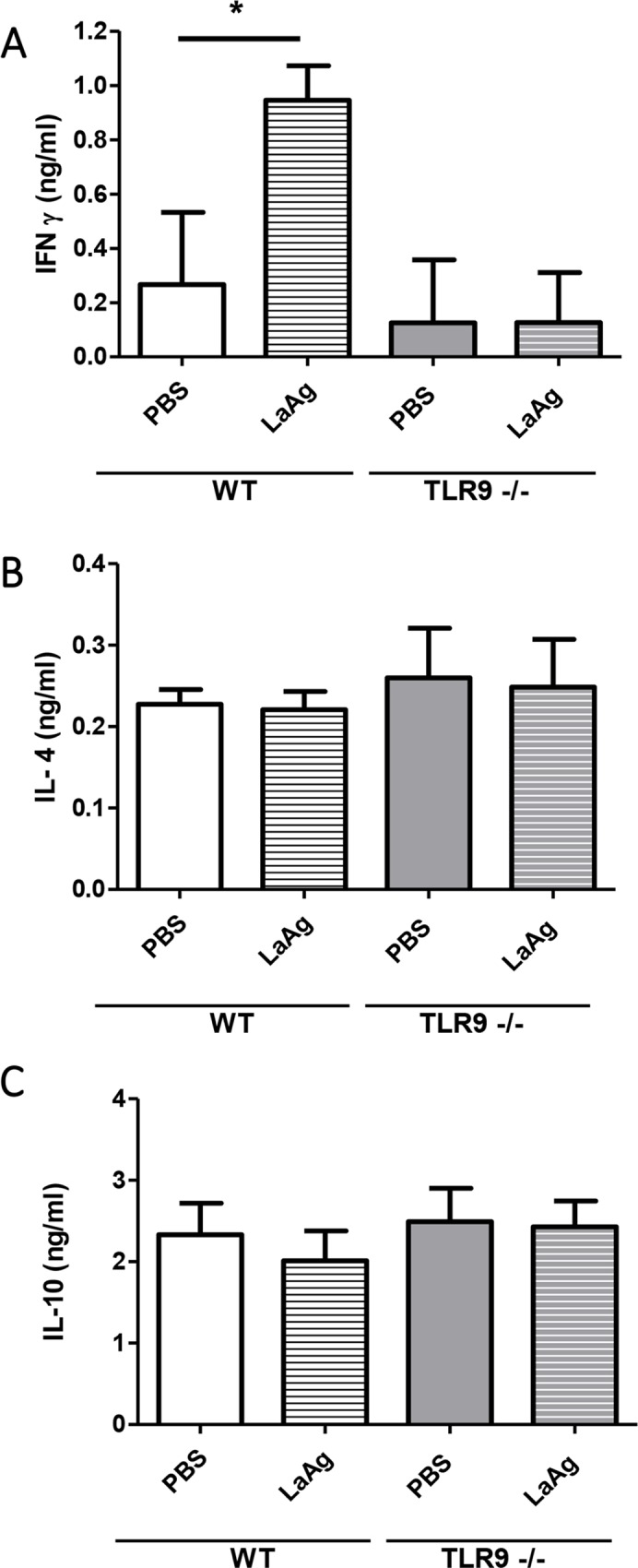
Cytokine production in vaccinated WT and TLR9^-/-^ mice. Mice were vaccinated and infected as described. At day 3 of infection, the levels of IFN-γ (A), IL-4 (B) and IL-10 (C) were measured in the supernatants of LmAg-stimulated draining lymph node cells. (Mean ± SD; n = 5) *P≤0.05 in relation to PBS controls. Results representative of two independent experiments.

To investigate the lack of protection that controls the parasite load in vaccinated TLR9^-/-^ mice, the delayed hypersensitivity response after LaAg immunization and using the infection as challenge was evaluated. Vaccinated WT mice showed a strong delayed hypersensitivity response at 18 h, 21 h and 24 h, compared with control WT mice injected with PBS, control TLR9^-/-^ mice and LaAg vaccinated TLR9^-/-^ mice (Fig 12). Vaccinated TLR9^-/-^ mice presented a small but significant increase in thickness compared to the control TLR9^-/-^ mice only 21 h after challenge. Then, 3 days after infection, the draining lymph nodes were antigen-stimulated *ex vivo*. In WT mice, those that were vaccinated were able to produce IFN-γ (Fig 13A); however, the same was not observed in TLR9^-/-^ mice. No difference was observed in the production of IL-4 (Fig 13B) and IL-10 (Fig 13C). Taken together, we observed that vaccinated WT mice were more competent to induce cellular response in comparison with vaccinated TLR9^-/-^ mice.

## Discussion

TLRs serve as the first line of defense for innate immune cells and are important for protecting against *Leishmania* infections [8–16]. The importance of TLR signaling to protect against *Leishmania* infections is further established by studies that have as direct targets several TLRs using specific agonists and knockout mice [16]. It has already been described that TLR9 can be activated by non-methylated CpG DNA found in *Leishmania* [9], but not in mammalian cells, in which these sequences are normally methylated [10].

Neutrophils and macrophages are among the cells of the innate immune system that respond to *Leishmania* infection. These cells have several PRRs that help them to protect the host environment from invading pathogens. Thus, we decided to evaluate the *in vitro* infection by *L*. *amazonensis* in TLR9^-/-^ cells in comparison with WT C57BL/6 mice. Our results showed no significant difference between the infection of macrophages from TLR9^-/-^ and WT mice in addition to NO production (Fig 1A–1E). Likewise, the infection by *L*. *amazonensi*s did not induce formation of NETs (Fig 2A–2C). Although other works analyzed the role of TLR9 in different *Leishmania* species [12,14, 26, 27], none of them examined the *in vitro* macrophage infection and neutrophil NET formation induced by *L*. *amazonensis*. We also evaluated the infection of dendritic cells by *Leishmania amazonensis*, however, it was not possible to observe the activation of infected dendritic cells in WT and TLR9^-/-^ (Fig 3). Although *L*. *amazonensis* has the ability to impair DC activation and it is not related to TLR9. Indeed, the same phenotype was observed before on dendritic cells from WT infected with *L amazonensis* [22, 23, 24].

To evaluate the role of the TLR9 receptor *in vivo*, WT mice and TLR9^-/-^ mice were infected with *L*. *amazonensis*. The parasite load 7 days post-infection showed no difference between the groups (Fig 4), indicating the lack of an early response dependent on TLR9. The groups also presented a similar profile of lesion progression during the infection (Fig 5A–5D). However, the TLR9^-/-^ mice showed a slight increase in the lesion size at the peak of infection, which coincided with an increase in parasite load (Fig 5). These results are similar to those found in studies with *Leishmania major* [12], *Leishmania braziliensis* [14], *Leishmania infantum* [28] and *Leishmania guyanensis* [27]. These results suggest the participation of TLR9 in the immune response against leishmaniasis.

Antibodies have a pathogenic role in infection by *L*. *mexicana* [29, 30] since knockout mice for antibody receptors are able to resolve infection against those parasites. IgG1 antibodies are considered the pathogenic antibodies in *L*. *mexicana* infection [30]. Antibody production is also associated with pathogenesis in the *L*. *amazonensis* model, since BALB/JhD mice, a lineage that lacks B cells, is more resistant to infection [31] or using BALB/XID mice [32]. For this reason, we analyzed the levels of serum immunoglobulins of both WT and TLR9^-/-^ groups. TLR9^-/-^ mice produced higher amounts of IgG and IgG1 when compared with WT mice (Fig 6B and 6C), but the levels of IgM were similar between these groups (Fig 6A). In the first moment, we expected to observe the reduction of IgG and IgG1 in TLR9^-/-^ may be due to direct effect on B cells since the importance of TLR9 to activation and production of antibody [33, 34] that was observed in *L donovani* infection when endossomal TLR^-/-^ mice (Unc931bLetr/Letr mice) presented a reduction of IgG production [35]. However, we observed an increase of specific antibodies IgG in TLR9^-/-^ that corroborate with a recently finding that demonstrate that TLR9 activation inhibits proliferation, differentiation and production of IgG production of Follicular B cells [36]. Besides, the lack of TLR9 was directly related with the increase of IgG1 in other models [37, 38] corroborating our data. We suggest a pathogenic role for the antibody production, since TLR9^-/-^ mice presented higher levels of IgG1 along with larger lesions and higher parasite loads, which corresponds with previous data [29, 30, 31, 32].

It is already known that TLR9 activation induces a Th1 type immune response [39]. The ability of CpG (antigen that activates TLR9) to induce Th1 polarization made it an interesting new target for treating allergy and infectious diseases [40]. Therefore, we analyzed the Th1 cytokine that could be involved in the infection and in the increase of parasite load and lesion size in TLR9^-/-^ mice. We observed a reduction of IFN-y in the infected tissue of TLR9^-/-^ mice (Fig 6A). Flow cytometric analysis evidenced that the lack of TLR9 decreases the production of IFN-γ only in CD8^+^ T cells (Figs 7A, 8A and 8B) during *L*. *amazonensis* infection. It has previously been demonstrated that TLR9 does induce IFN-γ producing CD8^+^ T cells [41]. In our model, the number of IFN-γ producing CD4^+^ T cells was similar between the groups (Fig 9A and 9B), indicating that they did not fail to induce Th1 response (only IFN-γ producing CD8^+^ T cells). Similar findings showing that the activation of TLR9 increases IFN-γ producing CD8^+^ T cells, but not Th1 cells, have already been reported [42], corroborating our data. In addition, no difference was observed in the production of IL-4 and IL-10 (Figs 7B, 7C, 8C, 8D, 9C and 9D).

The participation of IFN-γ modulated by TLR9 is still unclear, since infections caused by different species of *Leishmania* result in different profiles. In *L major*, the reduction of IFN-γ produced by NK, but not by CD4^+^ T cells, was associated with increased number of parasites pathogenesis [12]. However, in another study using *L*. *major*, the production of IFN-γ was also reduced in CD4^+^ T cells [11]. In both studies, the production of IFN-γ by CD8^+^ T cells was not investigated. Independently, in these two studies, IFN-γ was associated with protection. Furthermore, it was shown that, in the absence of TLR9, a decrease of lymph nodes hypertrophy was observed [43] that is directly related to the absence of effector cells. In *L*. *braziliensis*, a different phenotype with increased hypertrophy in TLR9^-/-^ was observed, which increased the number of IFN-γ producing CD4^+^ T cells and IFN-γ producing CD8^+^ T cells. In *L guyanensis*, the lack of TLR9 allowed an increase of the Th2 response [27]. In our study, we did not observe any modification in lymph node hypertrophy (SF4). The reduction of IFN-γ was observed in the lesion site and in IFN-γ producing CD8^+^ T cells. Recently, it was demonstrated that TLR9 expression is predominant in lesion of patients with *L*. *amazonensis* in comparison with *L*. *braziliensis* [44], this could be an explanation for the differences in the immunological profile between these species. Further studies are necessary to gain a complete understanding of the role of TLR9; however, in general, TLR9 is related to the production of IFN-γ by different cell types that is associated with the control of lesion size and parasite load.

The LaAg vaccine, which comprises the total lysate of *L*. *amazonensis*, contains fragments of *Leishmania* genomic DNA, proteins and other cell debris [4]. These DNA fragments, that contain CpG sequences, activate TLR9, thereby developing a protective immune response against infection. Our group has been studying the use of adjuvants to increase vaccine effectiveness in order to obtain a better combination promoting a greater effect of the vaccine against leishmaniasis [45]. Furthermore, the use of TLR9 agonists as mucosal vaccine adjuvants has been studied to understand the best way to activate this receptor and increase the immune response protection that it can trigger [38].

LaAg vaccine induced protection via the intranasal route [3], although it failed to induce protection by the intramuscular route [6,46]. As it was confirmed in this work, following vaccination and challenge with *L*. *amazonensis* the infection in the paw had a reduced lesion size compared to the unvaccinated group (Fig 10A), showing that the administration by intranasal route of the LaAg vaccine is systemically effective. The choice of the administration route is very important, since leishmaniasis is a disease with broad spectrum, present in all continents and has an increasing number of cases worldwide [7]. The use of the intranasal route is an easy-to-administer technique, which facilitates mass immunization campaigns, does not require sterile injection and, therefore, has no personnel training costs. This is an interesting prospect, since leishmaniasis is considered a neglected disease that affects several countries with limited financial resources and would improve vaccine coverage worldwide [39]. It is also evident that it increases treatment compliance for promoting a painless immunization.

Although the only approach to respiratory mucosal vaccination that successfully achieved commercial use is nasal immunization against influenza, the major challenge for nasal vaccines is still the lack of licensed nasal adjuvants to enhance mucosal and systemic immunogenicity [47, 48]. Recently, it was demonstrated that TLR9 is present in nasal mucosa [17, 41]. Based on this and on the presence of DNA content in LaAg vaccine, we decided to evaluate the involvement of TLR9 in intranasal efficacy. We demonstrated the partial involvement of TLR9 in LaAg vaccine in the lesion control and parasite load (Figs 10 and 11). Although some studies investigate the profile of the immune response during vaccination against leishmaniasis, this is mainly focused on IFN-γ and iNOS [49], and few studies investigate the participation of receptors, specially TLR9, in vaccination mechanisms against leishmaniasis. From what we know at present, this is the first report that associates the importance of TLR9 receptor in LaAg vaccine efficacy against leishmaniasis. We showed that intranasal immunization with LaAg is dependent on TLR9 to induce effector function demonstrated by an increase of delayed hypersensitivity (Fig 12) and production of IFN-γ (Fig 13) by *ex vivo* antigen stimulation only in vaccinated WT mice but not in TLR9^-/-^ mice. Cellular response [50] and production of IFN-γ [51] have already been associated with a mechanism to control *L*. *amazonensis* infection. The same mechanism was described in the efficacy of LaAg intranasal vaccine, respectively, cellular response evaluated by hypersensitivity [5] and production of IFN-γ, [5,45] which supports our data shown here.

Understanding the mechanism of action and efficacy of first generation vaccines is important to improve second generation vaccines. It has been demonstrated that the yellow fever vaccine YF-17D activates multiple TLRs, including TLR9 in plasmacytoid and myeloid DCs [52]. The typhoid fever and BCG vaccines, which carry the same formula since their creation in 1911 and 1921, respectively, have DNA as adjuvant to activate TLR9. It is clear that the use of DNA as adjuvant is not a novel mechanism and, more recently, other studies demonstrated the positive effects of DNA as adjuvant in vaccines [53, 54]. It was proven that the use of BCG-DNA as adjuvant enhances the immune response in three viral swine diseases and also in *Taenia solium* cysticercosis, where the BCG-DNA or CpG-ODN increases the levels of IgG2, IFN-γ, the percentage of CD8^+^ T cells and specific proliferation of peripheral blood mononuclear cells [55, 56]. However, plasmid DNAs vaccines work through mechanisms other than the TLR9; it has been shown that CpG motifs found in plasmids don’t significantly increase the TLR9 response in DNA vaccines with both TLR9^-/-^ and wild-type mice presenting antibody and cellular immune responses to DNA vaccine similar in terms of magnitude and type [57].

Adjuvants are substances capable of increasing and improving the quality of the immune response, and they are often present in the subunit vaccines. Some adjuvants currently used are TLR ligands, such as AS04 (a TLR4 activator) [58]. Furthermore, TLR9 agonists were described as highly effective adjuvants for viral vaccines, capable of inducing specific and functional antibody responses with low doses [54]. CpG adjuvants have been reported as being effective and safe for intranasal vaccines [55, 56]. Here, for the first time investigating the LaAg vaccine, TLR9^-/-^ mice were vaccinated and their immune responses were evaluated. Our study indicates that TLR9 is associated with the mechanism of protection of intranasal LaAg vaccine and suggests that the use of a TLR9 agonist could be considered an adjuvant by intranasal route against leishmaniasis. The participation of TLR9 in LaAg intranasal vaccine efficacy through induction of systemic IFN-γ production is the main contribution of this manuscript.

### Conclusion

In this work, we observed that TLR9^-/-^ animals are more susceptible to infection, presenting a larger lesion size in the peak of infection, and also an increase of parasite load in the peak of infection and in the chronic phase. Moreover, the effectiveness of the LaAg vaccine is partially dependent on the activation of the innate immunity receptor TLR9, mainly in the chronic phase where the absence of the TLR9 receptor lead to no protection in vaccinated mice.

## Supporting information

S1 Fig Gate strategy used for dendritic cells. Spleen cells from infected WT and TLR9^-/-^ mice were plated at 1x10^6^ per well, infected overnight or not with *L*. *amazonensis*-CFSE, and stained for flow cytometry to determine the frequency of MHCIIhi and CD86^+^ gated on CD11c^+^.
(TIF)

S2 Fig Gate strategy used for lymph node CD4^+^ and CD8^+^ cells. Lymph node cells from infected WT and TLR9^-/-^ mice were plated at 5x10^5^ per well and stained for flow cytometry to determine the percentage of IFN-γ^+^ or IL-10^+^ cells gated on CD4^+^ or CD8^+^ in CD3^+^.
(TIF)

S3 Fig Percentage of i*n vitro* infection of dendritic cells by *Leishmania amazonensis*. Spleen cells (1×10^6^) from C57BL/6 WT and TLR9^-/-^ mice were incubated with 5×10^6^ *L*. *amazonensis* promastigotes stained with CFSE, or incubated with medium as a control. After 24 h, cells were stained for CD11c+ (PerCP). Cells were gated on CD11c+ expression. Percentage of CFSE+ cells from WT and TLR9^-/-^ mice (mean ± SD; n  =  4).
(TIF)

S4 Fig Lymph nodes cellularity in different experiments. Lymph node cells of infected WT and TLR9^-/-^ mice were quantified using a Neubauer`s chamber. (mean ± standard deviation; n  =  4–5). Numbers of cells in the popliteal lymph nodes at the peak of infection from two independent experiments.
(TIF)
