# Supplementary figures and images for: The role of TLR9 on Leishmania amazonensis infection and its influence on intranasal LaAg vaccine efficacy
Source: PLoS Negl Trop Dis. 2019 Feb 25;13(2):e0007146. doi: 10.1371/journal.pntd.0007146 (PMC6405171; doi:10.1371/journal.pntd.0007146)

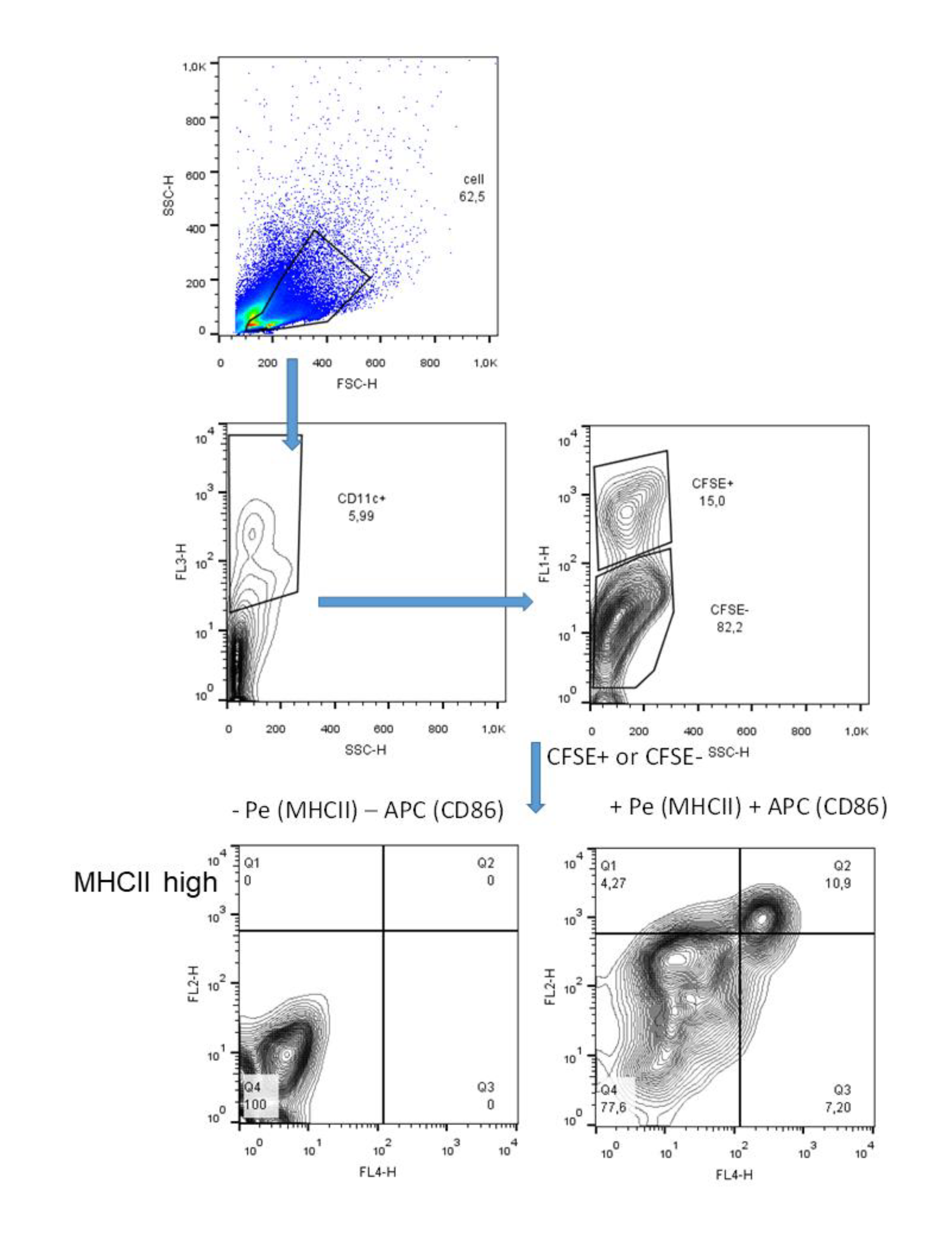

Supplement: S1 Fig — Spleen cells from infected WT and TLR9-/- mice were plated at 1x10^6^ per well, infected overnight or not with L. amazonensis-CFSE, and stained for flow cytometry to determine the frequency of MHCIIhi and CD86+ gated on CD11c+. (TIF) [file pntd.0007146.s001.tif]

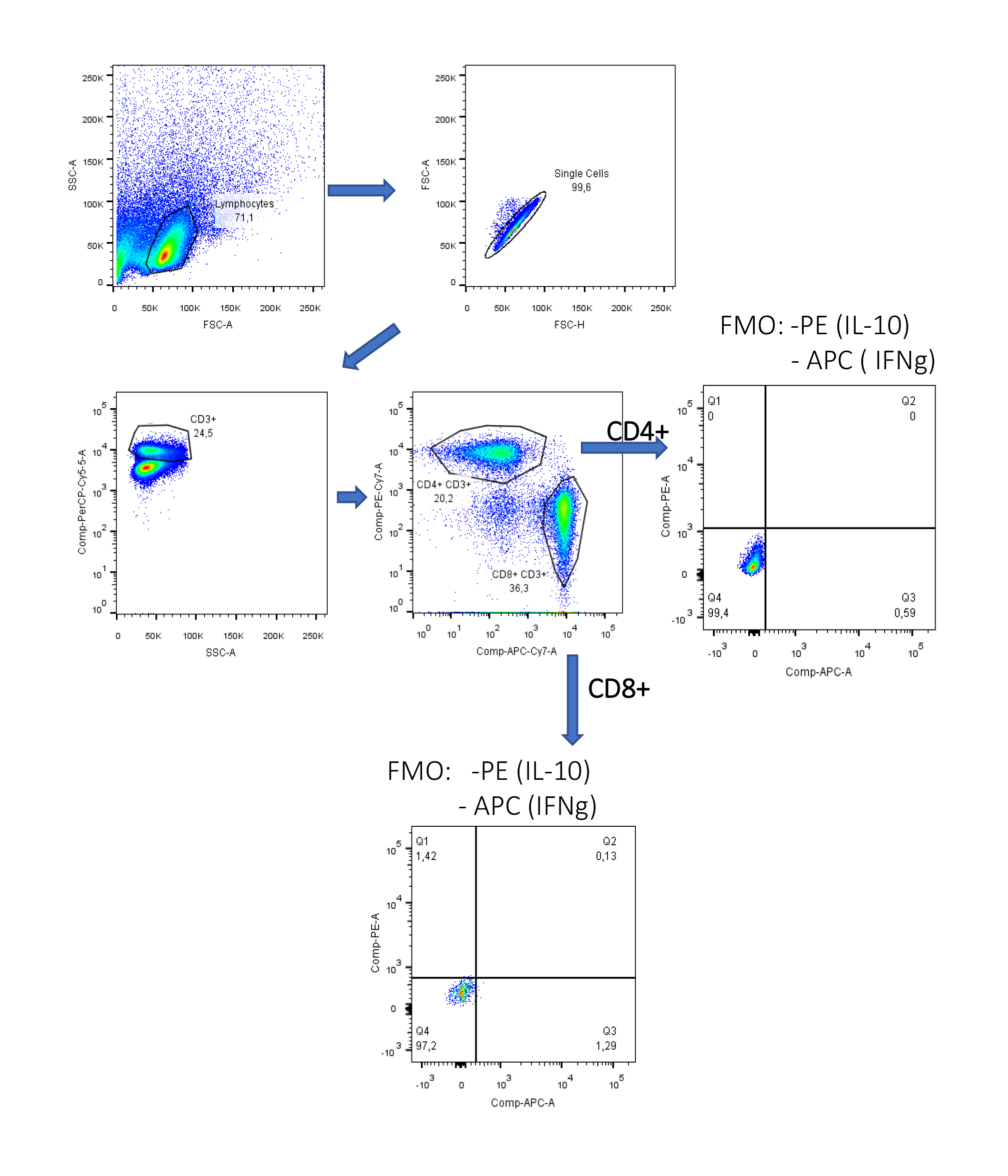

Supplement: S2 Fig — Lymph node cells from infected WT and TLR9-/- mice were plated at 5x10^5^ per well and stained for flow cytometry to determine the percentage of IFN-γ+ or IL-10+ cells gated on CD4+ or CD8+ in CD3+. (TIF) [file pntd.0007146.s002.tif]

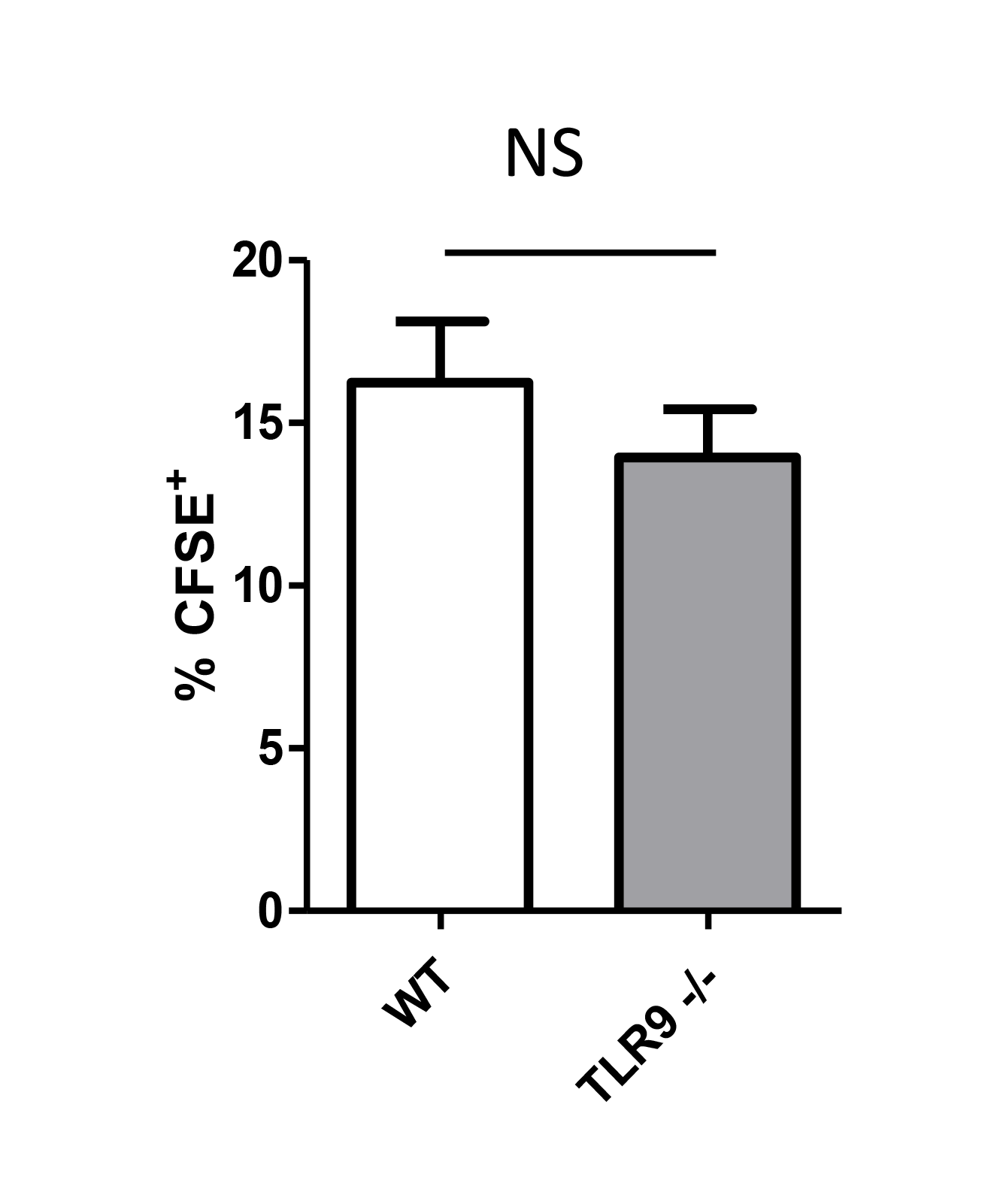

Supplement: S3 Fig — Spleen cells (1×10^6^) from C57BL/6 WT and TLR9-/- mice were incubated with 5×10^6^ L. amazonensis promastigotes stained with CFSE, or incubated with medium as a control. After 24 h, cells were stained for CD11c+ (PerCP). Cells were gated on CD11c+ expression. Percentage of CFSE+ cells from WT and TLR9-/- mice (mean ± SD; n = 4). (TIF) [file pntd.0007146.s003.tif]

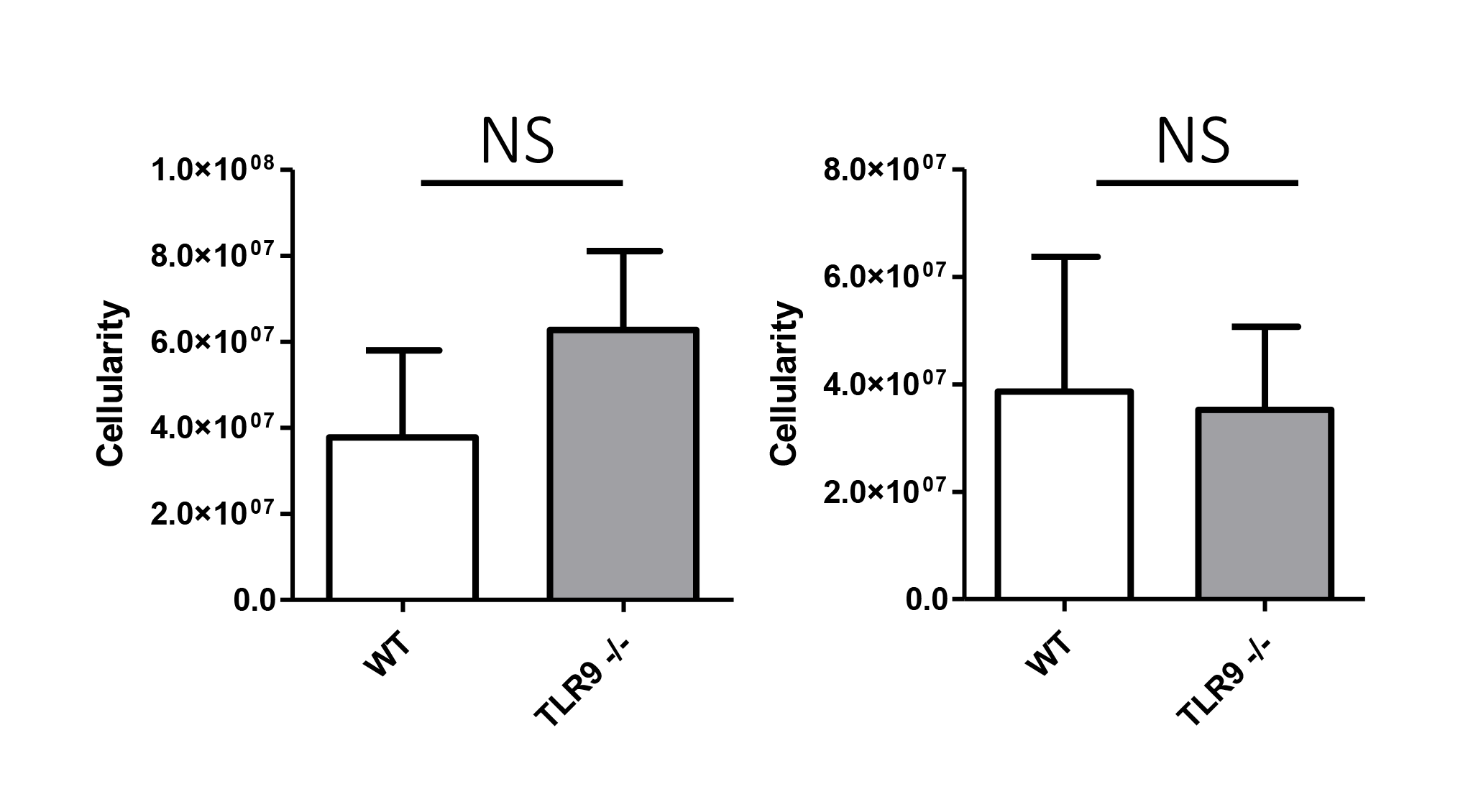

Supplement: S4 Fig — Lymph node cells of infected WT and TLR9-/- mice were quantified using a Neubauer`s chamber. (mean ± standard deviation; n = 4–5). Numbers of cells in the popliteal lymph nodes at the peak of infection from two independent experiments. (TIF) [file pntd.0007146.s004.tif]
